# Ionic Exchange Resins and Hydrogels for Capturing Metal Ions in Selected Sweet Dessert Wines

**DOI:** 10.3390/molecules23112973

**Published:** 2018-11-14

**Authors:** Gabriella Tamasi, Alessio Pardini, Claudia Bonechi, Alessandro Donati, Mario Casolaro, Gemma Leone, Marco Consumi, Renzo Cini, Agnese Magnani, Claudio Rossi

**Affiliations:** 1 Department of Biotechnology, Chemistry and Pharmacy, University of Siena, Via Aldo Moro 2, 53100 Siena, Italy; pardini4@student.unisi.it (A.P.); alessandro.donati@unisi.it (A.D.); mario.casolaro@unisi.it (M.C.); gemma.leone@unisi.it (G.L.); marco.consumi@unisi.it (M.C.); renzo.cini@unisi.it (R.C.); agnese.magnani@unisi.it (A.M.); claudio.rossi@unisi.it (C.R.); 2 Centre for Colloid and Surface Science (CSGI), University of Florence, Via della Lastruccia 3, 50019 Sesto Fiorentino, Firenze, Italy; 3 National Interuniversity Consortium of Materials Science and Technology (INSTM), Via G. Giusti 9, 50121 Firenze, Italy; 4 Operative Unit, University of Siena, CampoVerde, Calabria, Italy

**Keywords:** dessert wine, ionic exchange resin, hydrogels, metals, atomic absorption

## Abstract

Samples of sweet and dessert wines, Vin Santo (VSR) from Malvasia grapes, and Granello (GR) from Sauvignon grapes were collected and analyzed for the content of selected macro- and micro-nutrients (Na, K, Mg, Ca, Mn, Fe, Cu and Zn) and of Pb. GR wines had low levels for Fe, Cu and Zn, when compared to VSR and in particular Zn was two orders of magnitude lower. Methods to decrease the content of Zn and Cu in VSR, as well as those for reducing, at the same time, the concentrations of Ca, Mg and K in both VSR and GR, to avoid the formation of opalescence and depots of metal tartrates, were studied. Synthetic hydrogels containing l-histidine residue were tested. The overall relative lowering effects were by *ca* 4, 23, and 12% for K, Mg and Ca contents, and *ca* 6, 27 and 10%, for Mn, Cu and Zn contents, in GR wine samples. Commercial ion exchange resin Lanxess Lewatit L-207 and L-208 were then assayed, being legally allowed in the agro-food industry. The L-207 resin revealed great lowering effects on the concentrations of Mn, Cu and Zn, being 75, 91 and 97%, respectively, in VSR wines and 77, 76 and 92%, respectively, in GR wines. The content of Zn was reduced from 49.3 ± 1.2 mg/L in the original wine, down to 1.1 ± 0.1 mg/L, within 48 h soaking. The effects on the character of the dessert wines by the resin L-207 was also taken under control, measuring pH and color index. The color index changed by *ca* 15% and pH by *ca* 6% upon treatment of VSR wine with L-207 resins (48 h).

## 1. Introduction

Vin Santo (VSR) is a well known dessert wine produced and used mostly in Tuscany region (Italy) for several centuries, but even other regions in Italy contribute to the production of this beverage [1]. Furthermore, the European Community regulation by the year 2005 admits the usage of the term for certain dessert wine from the Santorini island in Greece [2]. It is obtained from grapes that, after harvesting, are aged for some two months in dark, well aerated and dustless rooms, at *ca* 18–20 °C. That is usually performed by storing the grapes over racks of layers of reeds, or hanging the bunches with wood or metal racks, so that the berries can lose most of the humidity content (drying step), without developing any significant amount of mold. The control of the main chemical parameters (like, pH, overall acidity) and of the stability of the product during the wine-making stages (refinement, storing and aging processes), are of great importance for preserving the quality of final products, as well as for economical aspects of the wine-making companies, and even for the territory. An amber color (for Vin Santo) or pale yellow (for Granello (GR), “passito” wine), excellent clearness, good brilliance, and a fresh and characteristic fruity smell are related to low pH values. Even the concentrations of cationic micro- and macro-nutrients like iron, copper and zinc, as well as potassium and calcium, and specific ranges for concentrations of anionic species, play important roles for the quality of those wines. It is well known that pH higher than 3.8–4.0 negatively influence on the color and aroma, and decreases the chemical and microbiological stability of wines [3,4,5,6]. Moreover, it is known, that wines at high pH cannot be corrected by addition of acids (i.e., tartaric acid, succinic acid) for several reasons: for example, the wine could already have a high tartaric acidity, and further addition of organic acid could allow the formation of potassium hydrogen tartrate (KHT) deposits, especially if the content of potassium is also high. Another undesired effect caused by high concentrations of tartaric acid is the sense of astringency. The usage of ionic exchange resins for the pH and acidity tuning, as well as for improving the tartaric and oxidative stability in wines, has been performed for decades, by using many kind of materials, spanning from commercial styrene-divinylbenzene copolymer gels, mostly containing functional groups of sulphonic acid [7,8,9,10], polyvinylimidazole and polyvinylpyrrolidone copolymers (PVI-PVP) [11,12], Dowex resins [13], chelating iminodiacetate [13], and carboxylmethylcellulose (CMC) [14] based materials. However, the resins that are commonly used by the wine-making companies, and currently accepted by law [15], are only PVI-PVP copolymers and CMC-based materials. In the case of the common divinylbenzene family resins, the supporting skeleton is functionalized with groups having high affinity towards cations and anions (i.e., if the resin is of the general type R…H^+^ and has good affinity for K^+^, Mg^2+^ and Ca^2+^ ions, it can be used to keep under control the content of those cations). Therefore, once the wine is passed through a column packed with, or stored on a bed of that type of resin, the liquid will experience a lowering of K^+^, Mg^2+^ and Ca^2+^ concentrations (and then a decreased risk for precipitation of corresponding metal hydrogen tartrate), but will also experience a significant lowering of pH that cannot be below 2.8. Furthermore, those processes might cause damage to certain wine components that contribute to defining the aroma, the color, or that are very important for the nutraceutical potentials (resveratrol, quercetin). These parameters have been considered and reported [8,9,13]. The ion exchange resins also need regeneration over time, for instance the general resin R…H^+^ could be regenerated by a solution of sulfuric acid or hydrochloric acid, at a proper concentration, and the liquid that comes from the regeneration processes should be properly treated before it can be disposed of. Moreover, the degradation and erosion of the resin during the treatment of the wine must be carefully monitored, in order to avoid significant pollution of the wine, by the species from the resin itself. Those methodologies are approved and currently performed in wine-making and refining procedures [4,5]. This work aimed also to investigate for the first time the usage of synthetic materials based on temperature-responsive hydrogels, carrying amphoteric units of l-histidine residues able to form complex heavy metal ions. This part of the work came out from the experience acquired by the research group on drugs, metal-based-drugs, nutraceuticals properties [16,17,18,19,20,21,22] and delivery systems, for embedding and releasing bioactive molecules, such as smart hydrogels, cross-linked polysaccharides, and liposomes [23,24,25,26].

## 2. Results

### 2.1. The Content of Selected Nutrient Metals in Granello (GR) and Vin Santo (VSR) Dessert Wines

The trend of the content for macronutrients C_Na_, C_K_, C_Mg_, and C_Ca_ in GR wine, during the time period 58th–110th days, from the sample collections (from the oak barrels at wine making company; Table 1, Figure S1a,b) showed that after 58 days in the laboratory conditions, the GR wine could be considered stabilized. The contents of the alkali metals C_Na_, C_K_, C_Mg_ and C_Ca_ averaged 28.3 ± 0.3, 754 ± 17, 200 ± 6, and 60 ± 3 mg/L at plateau underwent a significant decrease with respect to the initial values (7th day for GR samples; Table 1). The formation of opalescence and precipitate occurred during the stabilization time (before the 58th day), because the formation of insoluble species containing Na, K, and Ca. In fact, the concentrations of those metals decreased by 49, 11, and 66%, respectively. The contents of metal micronutrients C_Mn_, C_Fe_, C_Cu_ and C_Zn_ (Table 1, Figure S1c) revealed a small decrease for Mn (5%) down to the plateau value by 1.56 ± 0.02 mg/L, a decrease by at least 29% for Fe (0.28 ± 0.03 mg/L to below the limits of quantification (LOQ), 0.20 mg/L) and slight increases for Cu (15%) and Zn (2%) at plateau values by 0.39 ± 0.01 and 0.58 ± 0.02 mg/L, respectively. By contrast, the C_Mg_ increased significantly (*ca* 117%) from starting value (92 ± 7 mg/L) up to plateau value by 200 ± 6 mg/L. It can be noticed that the original sample bottles showed deposits and haziness, and during the two months in laboratory conditions, the entity of these phases changed markedly. That behavior would be reasonably interpreted as the occurring of chemical and biochemical processes towards an equilibrium state, meaning that some elements can increase and other decrease the total content in the final stabilized wine, on the basis of the specific solubility/stability, initial amount, kinetic of enzymatic reaction, of the single species. It is reasonable that Mg, which is an important co-factor for many types of enzymatic reactions, undergoes relevant changes.

The macronutrients data relevant to Vin Santo dessert wines (VSR) revealed a general trend similar to that, just above commented for GR wine (Table 2, Figure S2 and Table S2). In fact, the C_Na_, C_K_, and C_Ca_ in VSR wines underwent a general decrease during the stabilization time (before the 58th day), down to plateau averaged values, by 20.0 ± 0.5, 685 ± 24, 41 ± 4 mg/L, thus the lowering effects were 43, 12, and 32%, respectively, when based on initial values (7th day, 35.0 ± 0.5, 780 ± 28 and 61 ± 5 mg/L for C_Na_, C_K_ and C_Ca_, respectively). The most interesting data for micronutrients were those relevant to the content of Zn and Cu. In fact, their magnitudes at plateau 49.3 ± 1.2 (C_Zn_) and 2.68 ± 0.03 (C_Cu_) mg/L were much higher than for GR wines, and were also *ca* 25–100 times the values found for high-quality red wines from the same company [27], and higher than the maximum allowable concentrations by European and Italian laws (MAC_Zn_, 5 mg/L, MAC_Cu_, 1 mg/L) [28,29]). Even the C_Mn_, and C_Fe_ at plateau, were higher than those found for GR. Those facts had a rationale in the habit to use metal devices (iron wires or zinc-copper alloy wires) to hang up the bunches at the drying stage for the preparation of VSR. Beside those observations, the absolute values for C_Mn_ and C_Fe_ were below the maximum allowable concentrations by import regulation from China (MAC_Mn_, 2 mg/L and MAC_Fe_, 8 mg/L) [30] in both VSR and GR dessert wines and it is, therefore, assumed that those elements did not represent any threat for the quality of the products and safety of the consumers.

Finally, the content of Pb, C_Pb_, in the two dessert wines were 5.6 ± 0.4 (GR) and 15.7 ± 0.3 μg/L (VSR; Table S3), being in both cases, at least two orders of magnitude lower than the maximum allowable concentration enforced by European and Italian laws (MAC_Pb_, 150 μg/L) [31]. It was not considered necessary to perform any specific lowering strategy for that metal.

### 2.2. Assessing the Effect of Synthetic Hydrogels

**Scheme 1:**
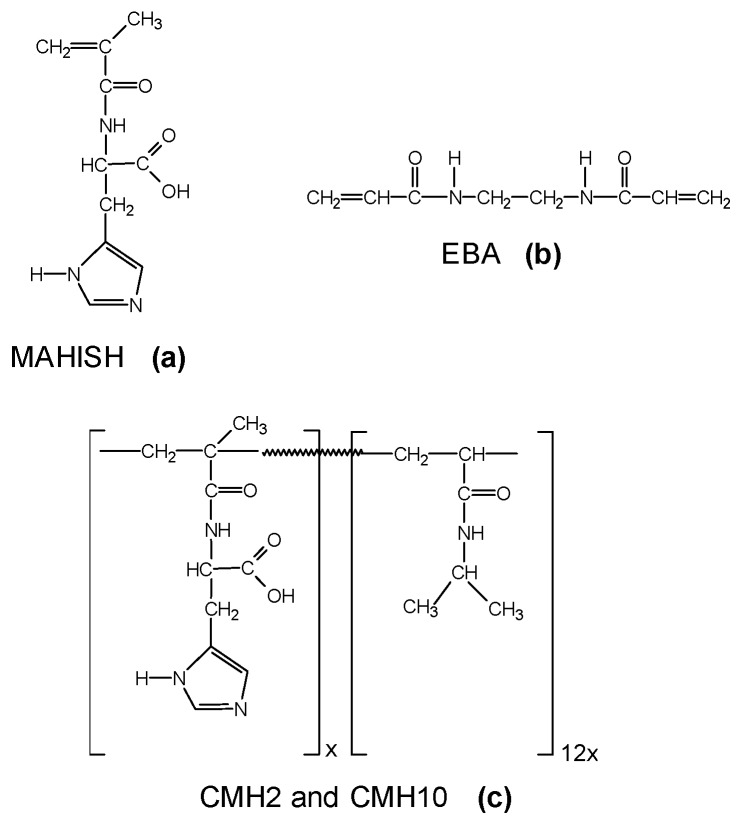
Structural formula for (**a**) monomer *N*-methacryloyl-l-histidine (MAHISH), (**b**) cross-linker, *N*,*N*’-ethylene-bis-acrylamide (EBA) and (**c**) general formula for linear polymers CMH2 and CMH10, poly(*N*-methacryloyl-l-histidine-co-*N*-isopropylacrilamide), NIPAAM/MHIST molar ratio 12, cross-linked with 2 and 10 mol% of EBA, respectively.

**Figure 1:**
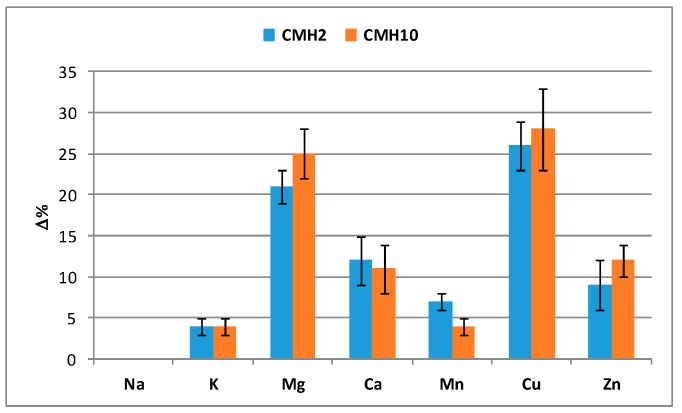
The lowering effects (Δ%) by swelled hydrogels on the content of selected metals in Granello (GR) wine (hydrogel/wine, 20 mg dry matter/10 mL, 2g/L; not stirred, in the dark, 20 ± 1 °C, 48 h). Values (mean ± ESD) obtained from three samples, and three replicates of each sample.

Once the content of each metal was considered constant, the assessments of effects by CMH2 and CMH10 hydrogels (Scheme 1) were performed on GR wines. On soaking the not swelled hydrogels (20 mg hydrogel (CMH2-nSW and CMH10-nSW) in 10 mL of GR wine) no appreciable lowering of metal content within 48 h was observed. In the case the histidine-based hydrogels (CMH2, CMH10) were previously swelled in ultrapure water and then soaked (48 h) into the GR wine (20 mg hydrogel/10 mL GR, 20 ± 1 °C, in the dark, not stirred systems) a lowering effect was revealed (Figure 1 and Table S4a,b). The data showed the following: (i) the effects on C_Na_ were null; (ii) the effects on C_K_ were small (4 ± 1%); (iii) the lowering effects on C_Ca_ and C_Zn_ were small (*ca* 10 ± 3%); (iv) the highest effects on C_Mg_ were 25 ± 3% and those on C_Cu_ were by 28 ± 5% (by using CMH10 material).

### 2.3. Assessing the Effect of Ion Exchange Resins

The subsequent step was that of determining the effects by solid state commercial resins of the ionic exchange type on samples of dessert wines. The selected commercial resins were Lewatit-207 (L-207) and Lewatit-208 (L-208), consisting of a matrix of polystyrene and divinylbenzene polymers with chelating functional groups based on iminodiacetate [32], able to capture mostly divalent metal cations (Cu, Pb, Ni, Zn, Cd, Fe, Be, Mn, Ca, Mg, Sr and Ba). It has to be noted that similar resins have been used for a long time in the food industry in several countries, including those within the European Union, for the treatments of waters for human uses, as water and fruit juice sweeteners, as acids for food uses, and other similar purposes [3,4,5,8,33,34].

**Figure 2:**
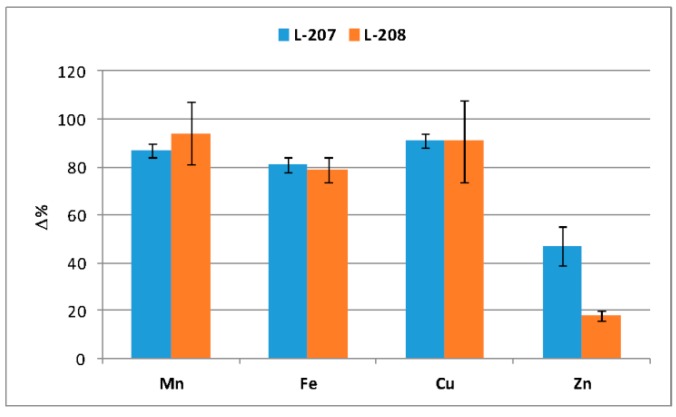
The lowering effects (Δ%) by the resins L-207 and L-208 on the concentrations of micronutrients in VSR wine (resin beds/wine 300 mg/15 mL, 20g/L; not stirred, in the dark, at 20 ± 1 °C, for 48 h). Values (mean ± ESD) obtained from three samples, and three replicates each sample.

A preliminary assay was carried out by using 300 mg of resin (L-207 or L-208; immersion beds, previously activated) on 15 mL of VSR samples. The data for the C_Mn_, C_Fe_, C_Cu_ and C_Zn_ in VSR wine are reported in Figure 2 (and Table S5a,b). Both the resins had large decreasing effects for C_Mn_ and C_Fe_ (*ca* 80–90%), and significant decreasing effects for C_Cu_ and C_Zn_, and L-207 had lowering effects larger than L-208 as regards C_Zn_.

### 2.4. Large Scale Study on L-207 Resin over Increasing Time

A larger scale experiment was carried out by using L-207 activated resin (20 g) for treating 1.00 L of wine sample (both VSR and GR). The data for the effects on the content of metals at different soaking times were recorded (the change of wine volume after each sampling, of 5 mL, was taken into account). The data are reported in Table 3 and Table 4 (see also Figure S3).

It is evident that the lowering of C_Mn_, C_Cu_ and C_Zn_ in GR was very effective. In fact, the C_Mn_ after 48 h treatment (resin bed *ca* 2.0% *w*/*v*, dark) was reduced to 23% of the initial value, and final C_Mn_ was as low as 0.36 ± 0.03 mg/L. The C_Cu_ in the same condition was also much reduced, to 24% of the initial value (0.39 ± 0.01 mg/L). In the case of C_Zn_ the lowering was even larger, the concentration decreased to 8% of the initial value for GR (0.58 ± 0.02 mg/L). The effect on the concentration of alkaline metals was small (C_Na_, lowering to 87%; C_K_, lowering to 85%) and is related to the low charge/surface ratio for the ions. The effects on C_Mg_ and C_Ca_ lowered by 57 and 54% based on untreated GR wine.

As regards VSR wine, it has to be recalled that C_Mn_, C_Fe_, C_Cu_ and C_Zn_ were higher than the corresponding ones in GR. In the case of Mn the difference between the initial contents C_Mn_, was limited: 1.79 ± 0.03 (VSR) *contra* 1.56 ± 0.02 mg/L (GR). The difference increased for the C_Cu_ and C_Zn_ values. The trend of the lowering ratio versus the charge/surface ratio was almost linear for the micronutrients in VSR, R^2^ = 0.895, on passing from Mn to Zn, when the oxidation status for Fe was assumed as +2; whereas the data for Fe^+3^ did not fit the trend, confirming that iron is reasonably in the reduced state. The relative lowering effects on C_Mn_, C_Fe_, C_Cu_ and C_Zn_ were down to 25, 15, 9, and 2%, respectively, of the initial values. It was confirmed that macronutrients Na, K, Mg and Ca had almost the same concentrations in VSR with respect to GR wines. The C_Na_, and C_K_ were reduced just down to about 95% of the initial values, whereas C_Mg_ and C_Ca_ were reduced down to 61 and 44% of the initial values, by L-207. Interestingly, C_Zn_ in VSR samples averaged 49.3 ± 1.2 mg/L, resulting *ca* 10 times higher than the maximum value allowed by the enforced regulation (MAC_Zn_, 5 mg/L) [29]. Therefore, the optimized treatments, just above those reported, allowed a lowering of the C_Zn_ down below the MAC_Zn_ value, within *ca* 3 h of immersion (of 20.0 g of L-207 resin per 1.0 L of wine under stirring at 20 °C in the dark). The lowering to below 2 mg/L is reachable after 6 h treatment under stirring at a plateau that had C_Zn_ 1.1 ± 0.1 mg/L.

### 2.5. Effects of L-207 on Wine Character: pH and Color Index Indicator Parameters

The values of pH and color index (CI) were selected as indicator parameters for checking the overall macroscopic quality of the wines, during the treatments (Table 5 and Figure S4).

The initial pH value for GR wine before treatment with L-207 resin was 3.47 ± 0.03, then a plateau by 3.32 ± 0.02 was reached after 6 h (decreasing by 0.15 units, 4.3%); whereas for VSR the shift was from 3.45 ± 0.03 for untreated wine to a plateau by 3.23 ± 0.02 (decreasing by 0.22 units, 6.4%).

The color index (CI) values for GR wine changed from an initial absorbance value by 0.437 ± 0.007, to a plateau by 0.366 ± 0.012 after 6 h treatment (decreasing by 0.071 Abs units, 16%). As regards VSR, the CI change from the initial value by 1.056 ± 0.014, was stabilized down to a plateau by 0.912 ± 0.009 Abs unit (decreasing by 0.144 Abs unit, 14%, in 6 h treatment).

## 3. Discussion

### 3.1. Stabilization of Metals in GR and VSR Dessert Wines

Selected macro- and micro-nutrients were analyzed on GR and VSR wine samples, from the sampling time up to stabilization, that occurred within *ca* 2 months. The changes of the metal contents for the samples taken from the wood casks are due to both thermal and microbiological processes, that occurred during the storage at laboratory conditions (20 ± 1 °C). The samples were not homogeneous, at the very collection time and had evident precipitates/haziness due to the possible presence of yeasts, metal-tartrates, -sulfides, -carbonates, etc. It has to be noticed that the C_Mn_ in high-quality red Chianti wines from the same company, as previously determined from this laboratory, and coming from harvests in 2001–2008, were in the ranges 2.03 ± 0.01–1.26 ± 0.01 mg/L [27]. The previously reported C_Fe_, C_Cu_ and C_Zn_ for top-quality red wines ranged 4.68 ± 0.05–1.03 ± 0.01 mg/L, 0.20 ± 0.02–trace (<0.100) mg/L, and 1.10 ± 0.04–0.53 ± 0.03 mg/L, respectively [27]. Therefore, a good agreement was detected between present work values for the GR and VRS dessert wines and previously studied red wines, even though some differences are evident, particularly for Cu and Zn (in VSR). The diversity of fermentation, aging and storing procedures, as well as large differences in grape varieties, but similarity about “terroir” played some role.

In conclusion the stabilization, at controlled temperature for both GR and VSR dessert wines had to be allowed, and immediately after that period and after the relevant analytical determinations, proper treatments for decreasing the content of certain metals could be performed. The bottling and commercialization could occur afterwards.

### 3.2. The Effect of Synthetic Hydrogels

Regarding the novel application of the synthetic hydrogels, it has to be noted that these materials are expensive in terms of working time and cost of synthesis, purification, swelling, and regeneration. Furthermore, the hydrogels had a non-negligible erosion (qualitatively estimated by *ca* 5% each step, even at rest) and are not approved yet for the food and beverage industry to the best of our knowledge*.* The relative amounts of hydrogels used in these preliminary tests were small (20 mg/10 mL, *ca* 0.2% *w*/*w*). The efficiency for Cu^2+^ decreasing was 26 ± 3 and 28 ± 5% for CMH2 and CMH10 hydrogels, respectively. Therefore, that route does not have to be discarded in principle, but is worthy of further research. The synthetic hydrogels used have never been tested for wine treatments, neither for research or industrial purposes, to our knowledge. It can be noticed that very recently a paper by Friedenberg and co-workers (2018) [11] reported the usage of polyvinylimidazole and polyvinylpyrrolidone copolymers (PVI-PVP) to reduce copper content in white wines. The latter materials have been reported for several years [4] and are actually also accepted by European regulation (since 2009 [15]), but they were different from the “smart” hydrogels presented in this work. These materials have a great application as carriers of organic molecules, metal-complexes, anions and cations in bio-systems [24,35,36,37]. The study of these materials could be expanded in future investigations and papers, possibly by: (1) using larger amounts of hydrogels, (2) optimizing the nature of the functional active groups, (3) optimizing reticulation percentage, (4) determining erosion effects, (5) determining effects on wine organoleptic properties, (6) measuring the toxicity of hydrogels. Of course, all of that is not possible in this paper. Finally, we wished to recall that this project managed to compare the effects of dry hydrogels and pre-swelled ones, because in case the dry materials would have worked, the potential industrial application could skip that step. Unfortunately, dry materials did not work and only water pre-swelled gels were able to decrease the content of certain metals.

### 3.3. The Effect of Ion Exchange Resins

The two commercial resins were efficacious, and were easier to manipulate than the synthetic hydrogels. Furthermore, the resin L-207 was considered more suitable than L-208, because of its lowering effect for C_Zn_. Based on these data, it was decided to extend the analysis of the effects by L-207, at larger scale in VSR and GR dessert wines. The results showed a great quenching for Cu^2+^ and Zn^2+^ that were the main focus of the work particularly in VRS wine. This was reached within a few hours of soaking for the resin under moderate stirring, to guarantee a limited erosion (that was below 2% each treatment) and possibly low changes of general flavors and other tasting properties. In fact, both VSR and GR wines underwent a moderate decrease of pH and color index upon treatments, suggesting that even the tastes of the products and the content of polyphenols and esters were not much altered by the study treatments. Finally, it is interesting to underline that these resins were already approved for the food and beverage industry. If a wine-making company treats *ca* 90 L of VSR, reduces the concentration of Zn from 49.3 ± 1.2 to 1.1 ± 0.1 mg/L, and mixes the new treated wine with *ca* 10 L of untreated wine, 100 L wine at C_Zn_ < 5 mg/L (MAC_Zn_) can be obtained. This imply that the flavor of the final product might be altered at lesser extent, with respect to the treatment of the full wine amount, and this allows also a lower resin and general handling working cost. That general strategy was considered better than constructing plants with columns packed with the resin. This latter should use a system of pumps and valves, that would add the risk of contamination of the wine through the contact of the product with mechanical devices.

Attention was also devoted to the ecological aspects related to the treatments of dessert wines with L-207 resin, requesting activation and regeneration processes. The waste materials are in fact aqueous acidic solutions, but their acidity and amount are small when compared to the amount of treated wine. Hypothesizing the treatment of 1 hL of wine, the usage of about 5 L of HCl (2M) activating solution, 10 L of water used for the washing, and 2 L of discarded acidified wine, can be predicted. These acidic solutions need to be neutralized by treating them with NaOH (*ca* 0.4 kg), before being discarded as waste (*ca* 15 L of salt solution). Furthermore, the turnover of the resin has to be considered, mostly because of erosion and reduced activity. On assuming an erosion by *ca* 2% each treatment cycle (1 hL), times 10 cycles (10 hL of treated wine), it means that less than 500 g of resin are predicted to be disposed to waste. This corresponds to 2000 bottles (0.5 L each) of the final product, ready for the market. The exhausted resin could be sent back to the resin producer for overall reconditioning.

## 4. Materials and Methods

### 4.1. Chemicals and Materials

Standard solution for atomic absorption analyses were purchased from Sigma-Aldrich (Milan, Italy) as TraceCERT single metal 1000 mg/L of Na, K, Ca, Mg, Mn, Fe, Cu, Zn, and Pb solution in 0.2% nitric acid. Suprapur nitric acid (65%), l-histidine, acryloyl chloride, *N*,*N*’-ethylene-bis-acrylamide (EBA), *N*-isopropylacrylamide (NIPAAM) were also purchased from Sigma-Aldrich (Milan, Italy). Ionic exchange resins Lewatit L-207 and L-208 were purchased from Lanxess (Colonia, Germany; Table 6). The two poly(*N*-methacryloyl-co-*N*-isopropylacrylamide) hydrogels (CMH2 and CMH10) were synthesized as previously reported [35,36,37]. In both cases, the NIPAAm/MHist molar ratio was 12 with different EBA (*N*,*N*’-ethylene-bis-acrylamide) cross-linking contents (2 mol%, CMH2; 10 mol% CMH10). The bidistilled water was produced by an Acquinity P/7 distiller (MembraPure GmbH, Berlin, Germany).

### 4.2. Dessert Wine Samples

As regards VSR dessert wine, Malvasia grape bunches that consisted of good quality healthy berries had been harvested from 12-year-old vines, late in the proper season *ca* half of September and had a high sugar content, that corresponded to a density of the grape juice by *ca* 1.11 g/mL. After drying (standing suspended in well ventilated rooms for several months) the bunches had been crushed lightly and fermented slowly, and then placed in French oak bariques (by 225 L). A subsequent aging period by five years, allowed the final alcohol content to rise up to 15.5 ± 0.2% *v*/*v* (as measured through the official density method [4], over at least three replicates on separate samples). The collected aged VSR wines from the oak casks, were stored in cork sealed bottles (six), in horizontal position, in the dark, at 20 ± 1 °C. They were then submitted to periodic analysis for the content of selected metals, up to stabilization.

As regards GR dessert wine from top quality bunches of Sauvignon grapes, were harvested the same period of VSR, were stored in a ventilated, dried and dehumidified room for 20 days. Then the berries were cooled down to 12 ± 1 °C and mildly pressed. The must was then fermented in steel containers for 30 days, at temperature by 12 ± 1 °C. Then the sweet wine was stored in cork-sealed bottles for six months, in the cellar. Finally, the sampled bottles (six) were stored as just reported for VSR, and submitted to periodic analysis of the content of selected metals, up to stabilization.

### 4.3. Color Index (CI) Determination

The absorbances at λ = 420 nm, that is the characteristic wavelength for white wines, were acquired for measuring the color index (CI) parameter, by using the Lambda 10 Perkin Elmer spectrophotometer equipped with a continuous flow quartz cuvette (10 mm optical path length, Perkin Elmer (Milan, Italy). All measurements were carried out in triplicate on three samples as such, without any pre-treatment, at 20 ± 1 °C.

### 4.4. pH Determination

Determinations for pH values were performed through a Crison pH-meter model 507 equipped with a glass combined electrode (Crison, Barcelona, Spain). The instrument was calibrated by using Crison buffer solutions at pH 7.00 ± 0.01 and 4.00 ± 0.01, before performing each series of measurements. All measurements were carried out in triplicate on three samples as such, without any pre-treatment, at 20 ± 1 °C.

### 4.5. Metal Analysis via Atomic Absorption Spectrophotometry (AAS)

The analyses for the metals Na, K, Ca, Mg, Cu, Fe, Mn, Zn and Pb were performed through the atomic absorption spectrophotometry technique with thermal activation (flame, FAAS; AAnalyst300 spectrophotometer, Perkin-Elmer, Waltham, MA, USA), and graphite furnace electro-thermal activation (GFAAS; Varian SpectrAA 220Z with Zeeman background correction spectrophotometer; Cernusco sul Naviglio, Milan, Italy). Multi-element hollow cathode lamps (Cu-Fe-Mn-Co-Cr-Ni), (Na-K), and (Ca-Mg-Zn) and mono-element (Pb) hollow cathode lamps were used (all from Perkin-Elmer). For FAAS analyses, the flame was fuelled by air/acetylene mixtures, optimized for each element to reach a better signal/noise ratio and better limits of quantification (LOQ) and limits of detection (LOD). In the case of GFAAS analyses, the graphite tubes were of pyrolytic type with integrated L’vov platform (Varian). The absorbance recorded for each sample was an average of 10 readings (FAAS) or three readings (GFAAS). The standard solutions for calibration purposes were obtained by diluting commercial mother solutions (1000 mg/L), with ultrapure 0.2% HNO_3_ (65%, Suprapur) solution. The external calibration method was commonly used in properly selected linearity range for each element. Selected samples were also analyzed via the standard addition method [38] to exclude errors from matrix effects, the differences between the data found by the two calibration methods resulting <5%. External calibrations showing correlation factors R^2^ > 0.990 were accepted for quantitative analyses (Table S10). Other details relevant to the analytical methods can be found from [27,39]. All the metal determinations were carried out in triplicate, on three bottles for each sample. The LOQ for the FAAS methods for the analyzed elements were Na 0.100, K 0.200, Mg 0.050, Ca 0.300, Mn 0.100, Fe 0.200, Cu 0.100, and Zn 0.050 mg/L, respectively; and for the GFAAS method for Pb LOQ was 1.0 μg/L (Table S10).

The contents of selected metal nutrients (C_Na_, C_K_, C_Mg_, C_Ca_, C_Mn_, C_Fe_, C_Cu_ and C_Zn_) were analyzed in GR and VRS dessert wines before any treatment, in the time period 58–110th days from the sampling at the wine-making company, in order to evaluate the stability of the metal parameters. A first analysis was also performed at 7th (GR) or 9th (VSR) day from the sampling, to estimate the initial conditions. After the removal of the original seal, a sampling of 2 mL was performed from each bottle (*n* = 3). The bottles were then properly re-sealed with their own cork, surrounded by parafilm and stored in a dark room at 20 ± 1 °C. The analyses were performed on the samples as such, or after proper dilution (if necessary). The same metals, were then analyzed in wine samples, after treatment with synthetic hydrogels or commercial ionic exchange resins (see below), to assess the lowering metal content ability of the materials.

### 4.6. Hydrogels

#### 4.6.1. Synthesis

Hydrogels were prepared as copolymers of the commercial *N*-isopropylacrylamide (NIPAAm) and the synthetic monomer *N*-methacryloyl-l-histidine, with a radical polymerization reaction in the presence of the cross-linking agent *N*,*N*’-ethylene-bis-acrylamide (EBA). The specific procedures were those previously reported [35,36,37]. The synthesized hydrogels are hereafter named CMH2, and CMH10. The formula for the monomer (*N*-methacryloyl-l-histidine, MAHISH), the cross-linker (*N*,*N*’-ethylene-bis-acrylamide, EBA), and for linear polymers CMH2 and CMH10 (poly(*N*-methacryloyl-l-histidine)-co-*N*-isopropylacrilamide, NIPAAM/MHIST molar ratio 12, cross-linked with 2 and 10 mol% of EBA, respectively; Scheme 1).

#### 4.6.2. Hydrogel Swelling

Several tests were carried out in order to determine the swelling properties of the hydrogels36-38]. On summarizing, aliquots of 20.0 ± 0.1 mg each, of dry CMH2 and CMH10, were swelled in 10 mL of ultrapure water for 48 h. Subsequently, the swelled material was taken off water, set on Whatmann filter paper to remove excess water, and finally weighed. The swelled gels averaged 65.6 ± 5.0 mg for CMH2 and 60.3 ± 5.0 mg for CMH10, respectively (on three replicates). Then, both dry gels and swelled gels were used for preliminary experiments on evaluating the lowering efficacy for metal contents in GR dessert wine. Each experiment was repeated in triplicate.

### 4.7. Metal Contents in Dessert Wines after Treatments by Synthetic Hydrogels and Commercial Resins

#### 4.7.1. Treatments by Synthetic Hydrogels

The not swelled (CMH2-nSW and CMH10-nsW) and swelled (CMH2 and CMH10) hydrogels (20 mg, dry weight) were set in 10 mL of GR wine sample and stored at 20 ± 1 °C, in the dark, not stirred, in hermetically closed glass vessels, for 48 h before the analyses. Then, an aliquot (5 mL) was taken off from the batch, possible solid materials were filtered off through Whatmann filtering paper, and the clear solution was analyzed for the contents of metals. Each experiment, from gel soaking through the analysis, was performed in triplicate.

#### 4.7.2. Treatments by Lewatit-207 and Lewatit-208 Resins

In preliminary tests, aliquots (15 mL) of clear stabilized VSR wine, were treated with the resin (300 mg; immersion beds). The resins were previously activated (by treating them with HCl 2M (15 mL for 2 h, under stirring), then filtered and subsequently rinsed twice with ultrapure water (1 h each, under stirring), and finally filtered and brought to dryness (in the air). Then, the beds were added to the wine sample in a sealed glass vessel (50 mL overall capacity, 4 cm diameter) equipped with a screwed plasticized cork to guarantee perfect closure, and maintained at rest (not stirred) for 48 h in order to reach equilibrium (20 ± 1 °C, in the dark). After that, the vessels were open and the surnatants were filtered and finally analyzed. Each experiment was repeated in triplicate.

#### 4.7.3. Large-Scale Treatments by Lewatit-207 Resin

The VSR and GR wines (1.00 L each) were set on beds of the activated resins (20.0 g, previously activated by 100 mL of HCl 2 M), in sealed glass vessels (1.5 L overall capacity, 10.6 cm diameter) equipped with a screwed plasticized cork to guarantee the perfect closure. The system was maintained under stirring through a magnetic bar (5.0 cm length, 0.5 cm diameter, iron bar covered with Teflon film, at 60 rpm; 20 ± 1 °C. in the dark, 48 h). Fractions (5 mL each) were collected after 1, 3, 6, 18, 21, 24, 30, 42 and 48 h. Each experiment was repeated in triplicates.

### 4.8. Statistical Data Treatment

Six bottles of both VSR and GR were sampled. All the experiments were carried out in triplicate and triplicate analyses were performed for all measurements. Mean values and estimated standard deviations (esd) were calculated by using Microsoft Office Excel 2007, implemented with regression analysis subroutine, and Origin Pro8 SR2, v.0891 (B891).

## 5. Conclusions

The work showed that the resin L-207 had a significant ability to lowering C_Zn_, and also C_Mn_, C_Cu_ and C_Ca_ that were high in a certain year’s harvest, at least working at the optimized experimental conditions. The content of Mn might be related to the particular nature of the soil for the study area that had a high content of Mn in that area, as confirmed by results previously found for wines produced by the same company [27]. Regarding C_Zn_, the high value found from the study samples had a rationale in the usage of zinc-plated racks for grape drying steps. The proposed remedy for the study production, was the treatment with L-207 resin. For future production the substitution of the metal racks with polyethylene racks is strongly advisable. Notwithstanding this, the final product would have to be also treated with L-207 for keeping other metals, like copper, manganese and calcium, under control. Finally, it will be important to test the possible changes on organoleptic properties, by using panels of tasters and sommeliers. The capturing effects on selected metal ions by ion exchange resins and hydrogels on table white wines and dry dessert white wines could also be tested, although the protocol is reasonably exportable and applicable to other kind of white wines.

## Figures and Tables

**Table 1:** Content of metal nutrients (C_Na_, C_K_, C_Mg_, C_Ca_, C_Mn_, C_Cu_, C_Zn_, as mg/L) in GR wines as function of time (days). Values for C_Fe_ < LOQ (0.20 mg/L).

Day	C_Na_	C_K_	C_Mg_	C_Ca_	C_Mn_	C_Cu_	C_Zn_
58th	28.4 ± 0.5	770 ± 22	205 ± 3	64 ± 5	1.59 ± 0.05	0.40 ± 0.02	0.58 ± 0.03
59th	28.6 ± 0.5	768 ± 19	200 ± 3	65 ± 4	1.58 ± 0.04	0.39 ± 0.03	0.58 ± 0.03
60th	28.2 ± 0.5	767 ± 19	206 ± 6	66 ± 3	1.60 ± 0.03	0.38 ± 0.02	0.58 ± 0.03
61th	28.1 ± 0.5	765 ± 18	201 ± 5	63 ± 5	1.56 ± 0.02	0.40 ± 0.03	0.59 ± 0.04
62th	28.0 ± 0.6	730 ± 16	201 ± 3	60 ± 3	1.55 ± 0.02	0.39 ± 0.03	0.57 ± 0.04
63th	28.1 ± 0.6	738 ± 15	207 ± 5	63 ± 4	1.57 ± 0.02	0.41 ± 0.01	0.59 ± 0.05
64th	28.0 ± 0.5	753 ± 10	204 ± 6	60 ± 3	1.56 ± 0.02	0.40 ± 0.04	0.58 ± 0.03
65th	28.2 ± 0.6	760 ± 23	194 ± 5	59 ± 3	1.54 ± 0.02	0.37 ± 0.03	0.60 ± 0.03
66th	28.3 ± 0.3	775 ± 11	192 ± 7	62 ± 2	1.56 ± 0.03	0.39 ± 0.01	0.59 ± 0.03
67th	28.0 ± 0.6	740 ± 14	186 ± 5	58 ± 3	1.56 ± 0.03	0.40 ± 0.01	0.63 ± 0.05
68th	28.3 ± 0.6	725 ± 14	202 ± 4	55 ± 2	1.56 ± 0.02	0.40 ± 0.02	0.58 ± 0.04
70th	28.4 ± 0.5	735 ± 14	197 ± 5	59 ± 2	1.57 ± 0.02	0.37 ± 0.03	0.56 ± 0.04
80th	28.7 ± 0.7	760 ± 14	199 ± 5	58 ± 2	1.55 ± 0.03	0.39 ± 0.03	0.60 ± 0.02
90th	28.4 ± 0.6	750 ± 15	202 ± 4	57 ± 5	1.54 ± 0.02	0.40 ± 0.03	0.59 ± 0.03
100th	29.3 ± 0.6	755 ± 12	200 ± 4	55 ± 3	1.56 ± 0.02	0.36 ± 0.03	0.57 ± 0.03
110th	28.5 ± 0.6	780 ± 17	210 ± 8	57 ± 6	1.56 ± 0.02	0.37 ± 0.02	0.57 ± 0.04
Average *	28.3 ± 0.3	754 ± 17	200 ± 6	60 ± 3	1.56 ± 0.02	0.39 ± 0.01	0.58 ± 0.02
7th	55.7 ± 3.6	847 ± 50	92 ± 7	177 ± 7	1.65 ± 0.08	0.34 ± 0.04	0.57 ± 0.05
Δ% *	−49	−11	117	−66	−5	15	2

Values (mean ± ESD) obtained from three samples, and three replicates each sample; full experimental data sets are reported in Table S1. * Average values calculated from 58th–110th data; Δ% Relative percentage change = [(Average (58th − 110th) − 7th)/7th] × 100.

**Table 2:** Contents of metal nutrients (C_Na_, C_K_, C_Mg_, C_Ca_, C_Mn_, C_Cu_, C_Zn_, as mg/L) in Vin Santo (VSR) wines as a function of time (days).

Day	C_Na_	C_K_	C_Mg_	C_Ca_	C_Mn_	C_Fe_	C_Cu_	C_Zn_
58th	20.5 ± 0.6	708 ± 18	175 ± 5	36 ± 3	1.80 ± 0.06	1.30 ± 0.07	2.68 ± 0.09	48.5 ± 4.6
59th	20.1 ± 0.5	607 ± 26	177 ± 5	40 ± 4	1.79 ± 0.03	1.33 ± 0.05	2.67 ± 0.11	49.9 ± 7.9
60th	20.2 ± 0.6	650 ± 18	176 ± 5	40 ± 3	1.78 ± 0.06	1.31 ± 0.07	2.72 ± 0.09	50.8 ± 4.6
61th	19.8 ± 0.3	695 ± 21	178 ± 5	38 ± 5	1.70 ± 0.03	1.28 ± 0.04	2.65 ± 0.13	49.1 ± 7.8
62th	19.5 ± 0.3	689 ± 25	180 ± 6	36 ± 2	1.75 ± 0.06	1.29 ± 0.05	2.71 ± 0.12	47.5 ± 4.7
63th	19.6 ± 0.3	695 ± 27	182 ± 5	36 ± 3	1.82 ± 0.06	1.32 ± 0.06	2.74 ± 0.13	50.5 ± 3.7
64th	19.2 ± 0.3	692 ± 20	175 ± 6	42 ± 5	1.80 ± 0.06	1.31 ± 0.04	2.67 ± 0.14	48.5 ± 2.9
65th	19.4 ± 0.4	688 ± 22	178 ± 6	45 ± 4	1.77 ± 0.07	1.34 ± 0.05	2.63 ± 0.15	48.7 ± 3.0
66th	19.6 ± 0.2	690 ± 16	174 ± 6	45 ± 2	1.79 ± 0.07	1.28 ± 0.04	2.66 ± 0.12	49.9 ± 3.6
67th	20.1 ± 0.6	695 ± 16	165 ± 10	44 ± 4	1.80 ± 0.05	1.30 ± 0.04	2.65 ± 0.16	51.0 ± 2.7
68th	20.6 ± 0.5	694 ± 27	170 ± 9	45 ± 4	1.81 ± 0.04	1.32 ± 0.05	2.65 ± 0.13	50.2 ± 2.9
70th	20.5 ± 0.3	687 ± 33	172 ± 3	44 ± 4	1.83 ± 0.04	1.27 ± 0.06	2.73 ± 0.12	47.3 ± 3.1
80th	20.7 ± 0.4	691 ± 27	173 ± 7	43 ± 3	1.79 ± 0.05	1.29 ± 0.06	2.65 ± 0.12	50.0 ± 2.6
90th	20.4 ± 0.5	694 ± 29	168 ± 5	41 ± 3	1.81 ± 0.06	1.30 ± 0.05	2.66 ± 0.16	47.7 ± 2.0
100th	20.5 ± 0.6	693 ± 26	169 ± 9	41 ± 2	1.78 ± 0.05	1.33 ± 0.06	2.64 ± 0.15	49.6 ± 3.7
110th	19.8 ± 0.4	689 ± 28	179 ± 8	41 ± 2	1.82 ± 0.04	1.27 ± 0.06	2.68 ± 0.13	49.6 ± 3.3
Average *	20.0 ± 0.5	685 ± 24	174 ± 5	41 ± 4	1.79 ± 0.03	1.31 ± 0.03	2.68 ± 0.03	49.3 ± 1.2
9th	35.0 ± 0.5	780 ± 28	155 ± 12	61 ± 5	1.76 ± 0.05	1.36 ± 0.08	2.77 ± 0.15	45.0 ± 2.6
Δ% *	−43	−11	12	−32	2	−4	−3	10

Values (mean ± ESD) obtained from three samples, and three replicates each sample; full experimental data sets are reported in Table S2. * Average values calculated from 58th–110th data; Δ% Relative percentage change = [(Average (58th − 110th) − 9th)/9th] × 100.

**Table 3:** Content of metal nutrients (C_Na_, C_K_, C_Mg_, C_Ca_, C_Mn_, C_Cu_, C_Zn_, as mg/L) in GR wines, as a function of soaking time (up to 48 h) on L-207 resin beds (resin beds/wine 20 g/L, under stirring, in the dark, 20 ± 1 °C).

Time	C_Na_	C_K_	C_Mg_	C_Ca_	C_Mn_	C_Cu_	C_Zn_
0	28.3 ± 0.3	754 ± 17	200 ± 6	60 ± 3	1.56 ± 0.02	0.39 ± 0.01	0.58 ± 0.02
1	29.2 ± 2.9	750 ± 26	137 ± 9	43 ± 1	0.63 ± 0.05	0.27 ± 0.03	0.31 ± 0.04
3	27.2 ± 2.9	702 ± 18	124 ± 3	38 ± 3	0.46 ± 0.03	0.20 ± 0.02	0.20 ± 0.02
6	28.8 ± 2.9	672 ± 24	120 ± 3	37 ± 2	0.40 ± 0.05	0.15 ± 0.01	0.12 ± 0.02
18	27.5 ± 3.5	706 ± 17	117 ± 3	34 ± 2	0.37 ± 0.04	0.13 ± 0.01	0.093 ± 0.006
21	25.8 ± 3.4	743 ± 20	116 ± 1	33 ± 3	0.37 ± 0.04	0.12 ± 0.02	0.086 ± 0.006
24	25.8 ± 3.4	678 ± 17	115 ± 3	33 ± 4	0.36 ± 0.02	0.11 ± 0.01	0.081 ± 0.007
30	26.0 ± 3.7	684 ± 16	114 ± 2	33 ± 3	0.36 ± 0.02	0.10 ± 0.02	0.069 ± 0.008
42	24.5 ± 4.1	707 ± 15	114 ± 2	32 ± 4	0.36 ± 0.02	0.098 ± 0.010	0.058 ± 0.005
48	24.9 ± 3.2	643 ± 20	113 ± 2	32 ± 3	0.36 ± 0.03	0.095 ± 0.009	0.047 ± 0.008

Values for t = 0 are those from untreated wines. Values (mean ± ESD) obtained from three samples, and three replicates for each sample; the full experimental data sets are reported in Table S6.

**Table 4:** Content of metal nutrients (C_Na_, C_K_, C_Mg_, C_Ca_, C_Mn_, C_Fe_, C_Cu_, C_Zn_, as mg/L) in VSR wines, as a function of soaking time (up to 48 h) on L-207 resin beds (resin beds/wine 20 g/L, under stirring, in the dark, 20 ± 1 °C).

Time	C_Na_	C_K_	C_Mg_	C_Ca_	C_Mn_	C_Fe_	C_Cu_	C_Zn_
0	20.0 ± 0.5	685 ± 24	174 ± 5	41 ± 3	1.79 ± 0.03	1.31 ± 0.03	2.68 ± 0.03	49.3 ± 1.2
1	20.1 ± 2.2	648 ± 20	138 ± 3	23 ± 2	0.68 ± 0.10	0.50 ± 0.07	1.10 ± 0.11	5.24 ± 1.42
3	21.0 ± 1.8	690 ± 18	128 ± 2	18 ± 1	0.53 ± 0.04	0.40 ± 0.05	0.89 ± 0.14	2.07 ± 0.16
6	19.7 ± 2.3	682 ± 18	123 ± 1	17 ± 2	0.47 ± 0.04	0.34 ± 0.03	0.67 ± 0.12	1.26 ± 0.06
18	20.6 ± 2.9	674 ± 19	128 ± 4	17 ± 2	0.47 ± 0.03	0.28 ± 0.02	0.41 ± 0.04	1.21 ± 0.04
21	20.5 ± 2.8	666 ± 17	125 ± 2	18 ± 1	0.47 ± 0.03	0.26 ± 0.02	0.35 ± 0.03	1.06 ± 0.05
24	20.1 ± 2.3	649 ± 22	119 ± 3	18 ± 2	0.45 ± 0.02	0.23 ± 0.03	0.35 ± 0.02	1.34 ± 0.06
30	20.6 ± 2.7	686 ± 20	122 ± 3	17 ± 2	0.44 ± 0.03	0.21 ± 0.03	0.31 ± 0.02	1.00 ± 0.06
42	20.3 ± 2.4	681 ± 19	116 ± 2	18 ± 1	0.47 ± 0.03	0.20 ± 0.03	0.25 ± 0.02	0.94 ± 0.06
48	19.4 ± 3.0	655 ± 26	107 ± 2	18 ± 1	0.45 ± 0.04	0.20 ± 0.02	0.25 ± 0.02	1.06 ± 0.08

Values for t = 0 are those from untreated wines. Values (mean ± ESD) obtained from three samples, and three replicates for each sample; the full experimental data sets are reported in Table S7.

**Table 5:** Trend for pH and color index (CI; Abs, λ 420 nm) values in GR and VSR wines as a function of soaking time (up to 48 h) on L-207 resin (resin beds/wine 20 g/L, under stirring, dark, 20 ± 1 °C).

Time	GR		VSR	
	pH	CI (Abs Units) *	pH	CI (Abs Units) *
0	3.47 ± 0.03	0.437 ± 0.007	3.45 ± 0.02	1.056 ± 0.014
6	3.32 ± 0.02	0.366 ± 0.012	3.24 ± 0.03	0.912 ± 0.009
18	3.33 ± 0.02	0.372 ± 0.012	3.24 ± 0.02	0.903 ± 0.009
24	3.32 ± 0.03	0.367 ± 0.007	3.23 ± 0.02	0.906 ± 0.008
36	3.31 ± 0.02	0.364 ± 0.006	3.21 ± 0.02	0.901 ± 0.007
48	3.32 ± 0.01	0.366 ± 0.008	3.22 ± 0.02	0.902 ± 0.006

Values for t = 0 are those from untreated wines. Values (mean ± ESD) obtained from three samples, and three replicates each sample; the full experimental data sets are reported in Tables S8 and S9. * Data collected from wine samples treated with regenerated L-207 resin.

**Table 6:** General characteristics and selected recommended operating conditions, of the commercial resins, Lewatit-207 (L-207) and Lewatit-208 (L-208) (Lanxess, Germany). Data taken from product information.

	Lewatit-207 (L-207)	Lewatit-208 (L-208)
Appearance	Beige, opaque	Beige, opaque
Matrix	Cross-linked polystyrene	Cross-linked polystyrene
Functional group	Iminodiacetic acid	Iminodiacetic acid
Structure	Macroporous	Macroporous
Ionic form, as shipped	Na^+^	Na^+^
Particle size	0.61 ± 0.05	0.65 ± 0.05
Particle geometry	Sphere	Sphere
Density	1.10 g/mL	1.16 g/mL
Water retention	55–60%	58–64%
Total capacity (H-form)	min 2.0 eq/L	min 2.5 eq/L
Operating temperature	max 80 °C	max 80 °C
Operating pH range	1.5–9	2–12
Regenerant	HCl 7.5%(*w*/*w*) or H_2_SO_4_ 10% (*w*/*w*)	HCl 10% (*w*/*w*)
Conditioning	NaOH 4% (*w*/*w*)	NaOH 4% (*w*/*w*)
Rinse water requirement	5 times total bead volume	5 times total bead volume

## Supplementary Materials

Supplementary data associated with this article can be found online.
