# Supplementary material for: Ionic Exchange Resins and Hydrogels for Capturing Metal Ions in Selected Sweet Dessert Wines
Source: Molecules. 2018 Nov 14;23(11):2973. doi: 10.3390/molecules23112973 (PMC6278320; doi:10.3390/molecules23112973)
Supplement: Supplementary file 1 [file molecules-23-02973-s001.pdf]

Ionic exchange resins and hydrogels for capturing metal ions in selected sweet dessert wines

Gabriella Tamasi,[1,2,*] Alessio Pardini,[1,2] Claudia Bonechi,[1,2,*] Alessandro Donati,[1,2] Mario Casolaro,[1] Gemma Leone,[1,3] Marco Consumi,[1,3] Renzo Cini,[1] Agnese Magnani,[1,3] Claudio Rossi[1,2,4]

[1]*Department of Biotechnology, Chemistry and Pharmacy, University of Siena, Via Aldo Moro 2, 53100 Siena, Italy.* [2]*Centre for Colloid and Surface Science (CSGI), University of Florence, Via della Lastruccia 3, 50019 Sesto Fiorentino, Firenze, Italy.* [3]*National Interuniversity Consortium of Materials Science and Technology (INSTM), Via G. Giusti 9, 50121 Firenze. Italy.* [4]*Operative Unit, University of Siena, CampoVerde, Calabria, Italy.*

* Correspondence: gabriella.tamasi@unisi.it; claudia.bonechi@unisi.it; phone, +39 0577 232123.

SUPPORTING INFORMATION

Figure S1. Contents of metal nutrients (mg/L) in GR wines as function of time (58th–110th days): (a) data C_{Na} and C_{Ca}, (b) data C_K and C_{Mg}, (c) data C_{Mn}, C_{Cu} and C_{Zn}; C_{Fe} was lower than LOQ (0.20 mg/L). The data show that after *ca* 60 days, under the laboratory conditions the GR wine is stable at least as regards the analyzed nutrient metals. Values (mean ± esd) obtained from three samples, and three replicates each sample.

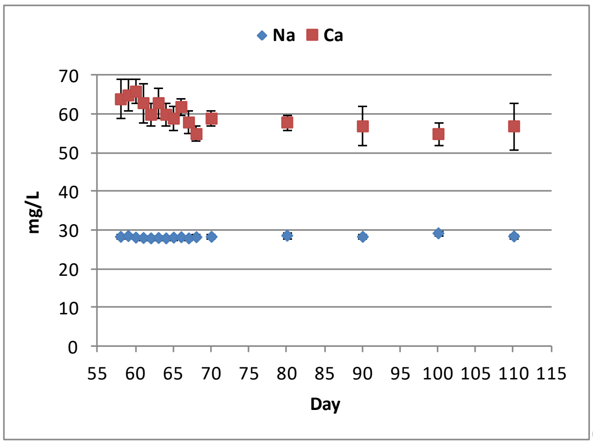

(a)

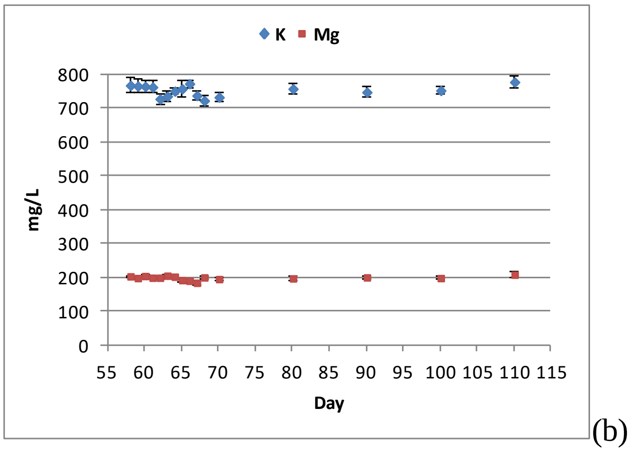

(b)

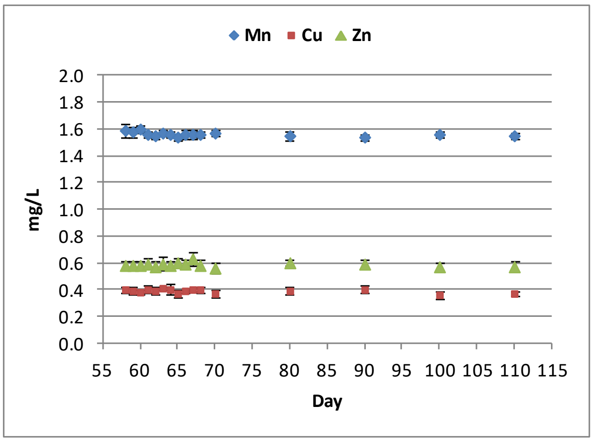

(c)

Figure S2. Contents of metal nutrients (mg/L) in VSR wine as function of time (58th–110th days): (a) data C_{Na} and C_{Ca}, (b) data C_K and C_{Mg}, (c) data C_{Mn}, C_{Cu} and C_{Fe}, (d) data C_{Zn}. The data show that after *ca* 60 days, under the laboratory conditions the VSR wine is stable at least as regards the analyzed nutrient metals. Values (mean ± esd) obtained from three samples, and three replicates each sample.

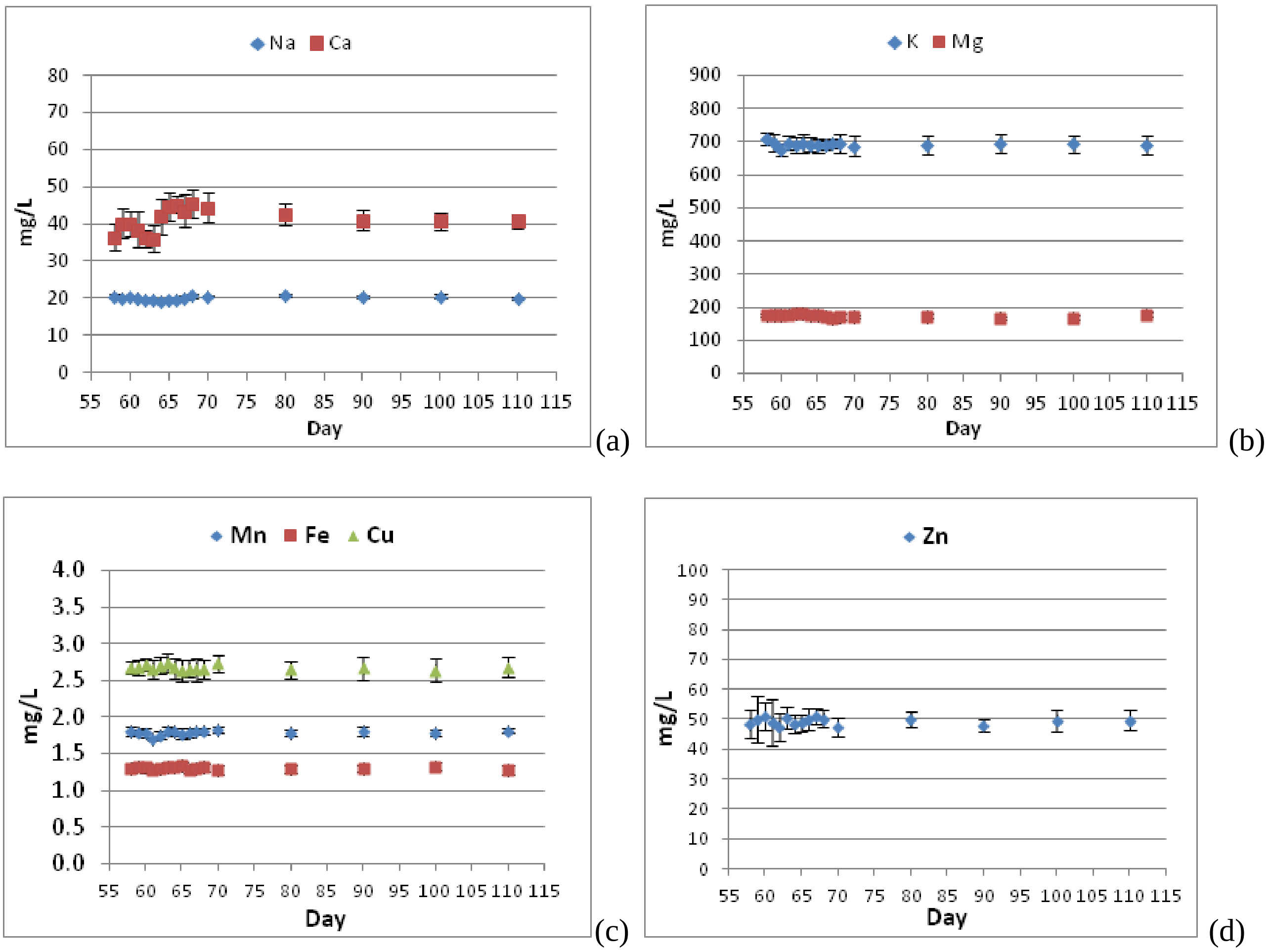

Figure S3. The effects by L-207 resin on (a) C_{Na} and C_{Ca}, (b) C_K and C_{Mg} and (c) C_{Mn}, C_{Cu} and C_{Zn} in GR wines, and (d) C_{Na} and C_{Ca}, (e) C_K and C_{Mg}, (f) C_{Mn}, C_{Fe} and C_{Cu}, and (g) C_{Zn} in VSR wines, as function of soaking time (up to 48 h; resin beds/wine 20 g/L, under stirring, in the dark, at 20±1°C). Values for t = 0 are those from untreated wines. Values (mean ± esd) obtained from three samples, and three replicates each sample.

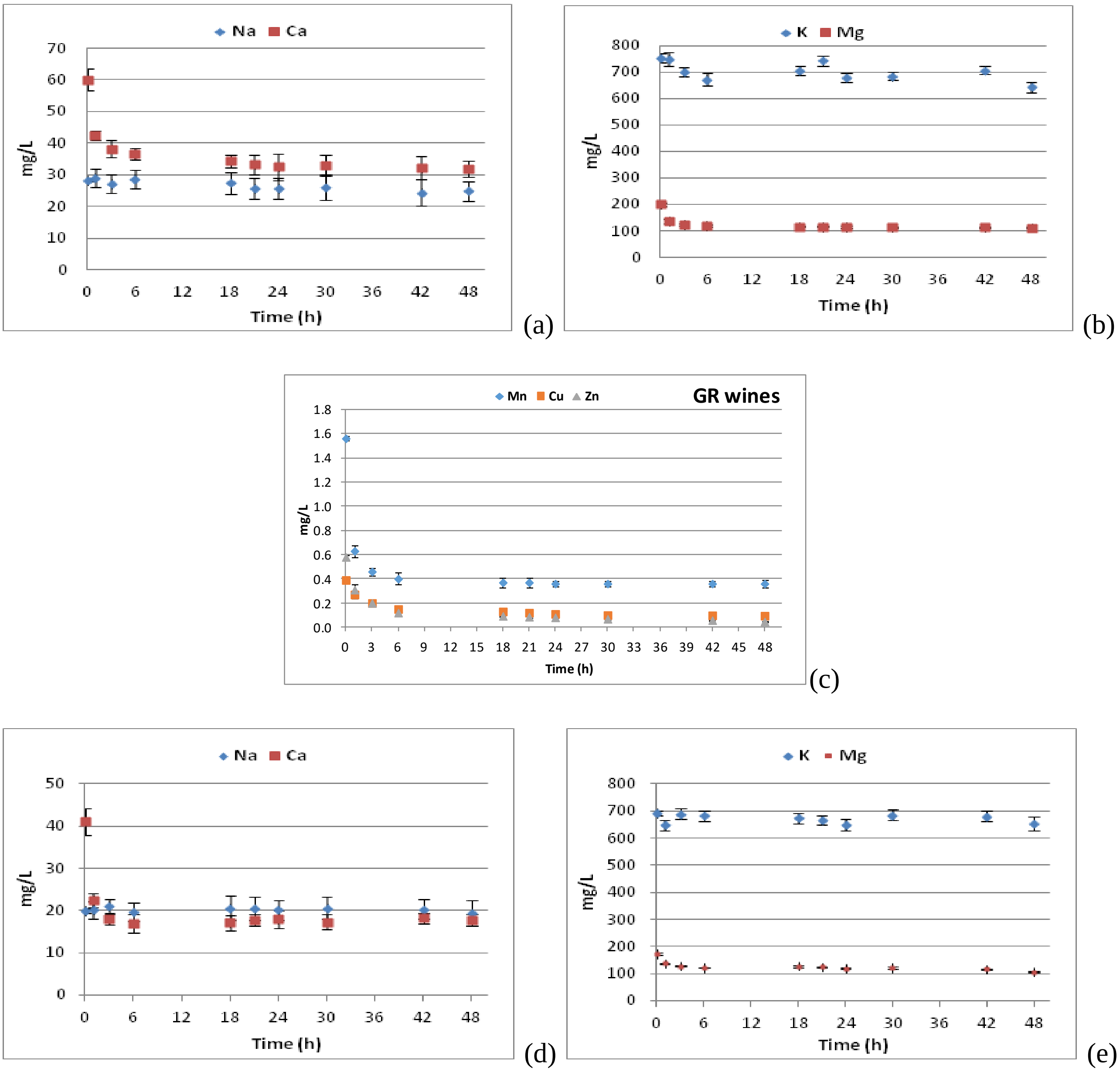

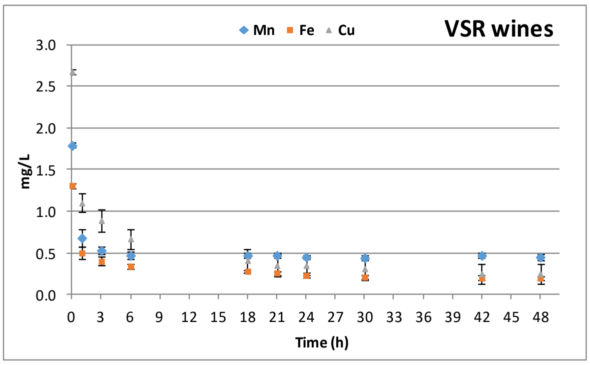

(f)

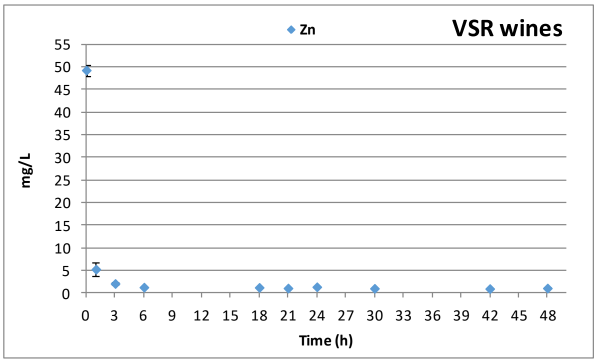

(g)

Figure S4. Effects on (a) pH and (b) color index (CI; Abs, λ = 420 nm) values *vs* soaking time (h) for GR and VSR wines on beds of L-207 resin (resin beds/wine 20 g/L, under stirring, in the dark, at 20±1°C). Values for t = 0 are those from untreated wines. Values (mean ± esd) obtained from three samples, and three replicates each sample. Color index values are from wines treated with regenerated L-207 resins.

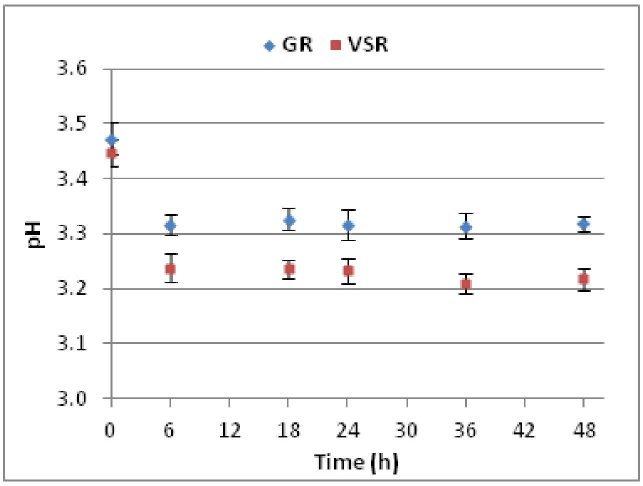

(a)

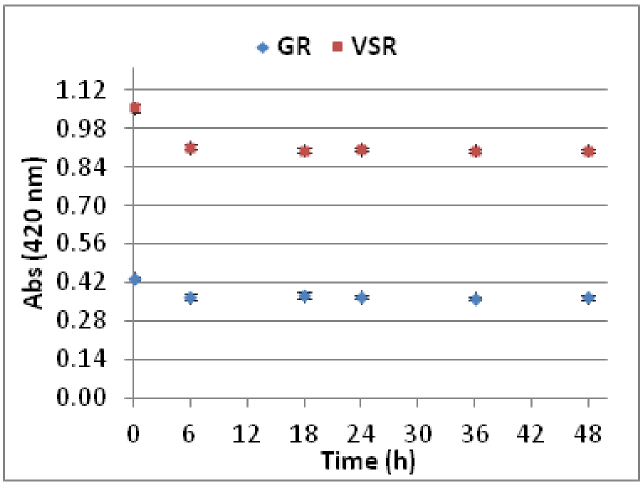

(b)

Table S1. Selected data of the content of selected metals (mg/L, Na, K, Ma, Ca, Mn, Cu, Zn) in GR wines as function of time (day) from the sampling at wine making company. The data for the three replicates.

Day	#1	#2	#3	Average	esd
**Na**					
**58th**	28.0	28.3	28.9	28.4	0.5
**59**	29.1	28.1	28.5	28.6	0.5
**60**	27.7	28.2	28.8	28.2	0.5
**61**	27.7	28.0	28.7	28.1	0.5
**62**	27.5	28.0	28.6	28.0	0.5
**63**	28.0	27.6	28.8	28.1	0.6
**64**	27.5	28.4	28.2	28.0	0.5
**65**	27.5	28.7	28.3	28.2	0.6
**66**	28.1	28.2	28.7	28.3	0.3
**67**	27.7	27.5	28.7	28.0	0.6
**68**	28.9	28.3	27.7	28.3	0.6
**70**	27.9	28.6	28.8	28.4	0.5
**80**	29.4	28.8	28.0	28.7	0.7
**90**	27.8	28.3	29.0	28.4	0.6
**100**	29.4	28.6	29.8	29.3	0.6
**110**	27.9	29.1	28.6	28.5	0.6
**Total Average**				**28.3**	**0.3**
**K**					
**58**	777	745	788	770	22
**59**	753	762	790	768	19
**60**	786	749	766	767	18
**61**	745	770	780	765	18
**62**	714	731	745	730	15
**63**	745	720	748	738	15
**64**	762	743	755	753	10
**65**	742	785	752	761	220
**66**	778	784	762	775	11
**67**	727	738	754	740	14
**68**	722	712	740	725	14
**70**	720	737	748	735	14
**80**	775	758	748	760	14
**90**	734	752	764	750	15
**100**	768	753	744	755	12
**110**	798	778	765	780	17
**Total Average**				**754**	**17**
**Mg**					
**58**	204	202	208	205	3
**59**	197	203	201	200	3
**60**	200	211	207	206	6

**61**	197	200	206	201	5
**62**	205	200	199	201	3
**63**	213	205	203	207	5
**64**	197	205	209	204	6
**65**	189	198	196	194	5
**66**	199	185	191	192	7
**67**	182	192	185	186	5
**68**	198	203	206	202	4
**70**	202	198	192	197	5
**80**	205	199	194	199	5
**90**	198	201	206	202	4
**100**	196	202	203	200	4
**110**	201	212	216	210	8
**Total Average**				**200**	**6**
**Ca**					
**58**	62	60	69	64	5
**59**	61	66	68	65	4
**60**	63	67	69	66	3
**61**	59	68	63	63	4
**62**	63	61	57	60	3
**63**	67	63	60	63	3
**64**	57	61	63	60	3
**65**	57	62	59	59	2
**66**	64	61	61	62	2
**67**	56	58	61	58	3
**68**	53	56	56	55	2
**70**	61	60	57	59	2
**80**	57	61	57	58	2
**90**	55	53	63	57	5
**100**	54	52	58	55	3
**110**	53	48	41	47	6
**Total Average**				**59**	**5**
**Mn**					
**58**	1.55	1.58	1.64	1.59	0.05
**59**	1.63	1.57	1.55	1.58	0.04
**60**	1.63	1.61	1.57	1.60	0.03
**61**	1.54	1.55	1.58	1.56	0.02
**62**	1.57	1.54	1.55	1.55	0.01
**63**	1.57	1.55	1.59	1.57	0.02
**64**	1.54	1.57	1.57	1.56	0.02
**65**	1.53	1.53	1.56	1.54	0.02
**66**	1.59	1.57	1.53	1.56	0.03
**67**	1.56	1.53	1.58	1.56	0.02
**68**	1.55	1.58	1.54	1.56	0.02
**70**	1.58	1.54	1.58	1.57	0.02

**80**	1.53	1.53	1.58	1.55	0.03
**90**	1.52	1.55	1.56	1.54	0.02
**100**	1.56	1.54	1.57	1.56	0.01
**110**	1.58	1.55	1.55	1.56	0.02
**Total Average**				**1.56**	**0.02**
**Cu**					
**58**	0.38	0.40	0.41	0.40	0.02
**59**	0.42	0.37	0.38	0.39	0.03
**60**	0.36	0.39	0.40	0.38	0.02
**61**	0.43	0.39	0.38	0.40	0.03
**62**	0.39	0.41	0.36	0.39	0.02
**63**	0.42	0.40	0.40	0.41	0.01
**64**	0.36	0.43	0.42	0.40	0.04
**65**	0.34	0.36	0.40	0.37	0.03
**66**	0.40	0.38	0.38	0.39	0.01
**67**	0.39	0.39	0.41	0.40	0.01
**68**	0.38	0.42	0.41	0.40	0.02
**70**	0.40	0.37	0.35	0.37	0.02
**80**	0.36	0.38	0.42	0.39	0.03
**90**	0.37	0.41	0.43	0.40	0.03
**100**	0.34	0.34	0.39	0.36	0.03
**110**	0.39	0.37	0.36	0.37	0.01
**Total Average**				**0.39**	**0.01**
**Zn**					
**58**	0.61	0.57	0.55	0.58	0.03
**59**	0.56	0.57	0.62	0.58	0.03
**60**	0.55	0.60	0.59	0.58	0.03
**61**	0.63	0.60	0.55	0.59	0.04
**62**	0.54	0.62	0.56	0.57	0.04
**63**	0.64	0.55	0.59	0.59	0.04
**64**	0.55	0.58	0.61	0.58	0.03
**65**	0.63	0.58	0.58	0.60	0.03
**66**	0.59	0.62	0.57	0.59	0.02
**67**	0.68	0.61	0.59	0.63	0.05
**68**	0.57	0.63	0.55	0.58	0.04
**70**	0.52	0.57	0.60	0.56	0.04
**80**	0.63	0.59	0.59	0.60	0.02
**90**	0.58	0.56	0.62	0.59	0.03
**100**	0.58	0.60	0.54	0.57	0.03
**110**	0.62	0.55	0.54	0.57	0.04
**Total Average**				**0.58**	**0.02**

Table S2. Selected data of the content of selected metals (mg/L, Na, K, Ma, Ca, Mn, Cu, Zn) in VSR wines as function of time (day) from the sampling at wine making company. The data for the three replicates.

Day	#1	#2	#3	Average	esd
**Na**					
**58th**	19.8	20.7	20.9	20.5	0.6
**59**	20.6	19.9	19.7	20.1	0.5
**60**	20.5	19.9	20.1	20.2	0.6
**61**	19.9	20.1	19.5	19.8	0.3
**62**	19.8	19.2	19.6	19.5	0.3
**63**	19.9	19.5	19.4	19.6	0.3
**64**	18.9	19.3	19.5	19.2	0.3
**65**	19.8	19.1	19.3	19.4	0.4
**66**	19.4	19.8	19.7	19.6	0.2
**67**	20.1	20.6	19.5	20.1	0.6
**68**	20.0	20.8	20.9	20.6	0.5
**70**	20.2	20.6	20.8	20.5	0.3
**80**	21.0	20.3	20.7	20.7	0.3
**90**	20.1	20.1	20.9	20.4	0.5
**100**	19.8	20.7	20.9	20.5	0.6
**110**	19.9	20.2	19.4	19.8	0.4
**Total Average**				**20.0**	**0.5**
**K**					
**58**	710	690	725	708	18
**59**	609	580	632	607	26
**60**	632	655	664	650	18
**61**	698	715	673	695	21
**62**	714	687	665	689	24
**63**	700	720	666	695	27
**64**	670	695	710	692	20
**65**	712	685	668	688	22
**66**	672	695	703	690	16
**67**	711	680	695	695	15
**68**	690	670	723	694	27
**70**	656	685	719	687	31
**80**	716	696	662	691	27
**90**	667	690	724	694	29
**100**	698	717	665	693	26
**110**	693	715	660	689	28
**Total Average**				**685**	**24**
**Mg**					
**58**	170	180	176	175	5
**59**	182	178	172	177	5
**60**	176	181	172	176	5

**61**	172	182	180	178	5
**62**	187	179	175	180	6
**63**	186	182	177	182	4
**64**	169	178	179	175	5
**65**	171	182	180	178	6
**66**	168	173	180	174	6
**67**	176	164	156	165	10
**68**	180	169	162	170	9
**70**	174	173	168	172	3
**80**	173	179	166	173	6
**90**	169	173	163	168	5
**100**	177	170	160	169	8
**110**	181	170	185	179	8
**Total Average**				**174**	**5**
**Ca**					
**58**	31	34	36	34	2
**59**	40	44	36	40	4
**60**	41	35	44	40	2
**61**	44	37	34	38	5
**62**	38	34	36	36	2
**63**	37	32	39	36	3
**64**	47	41	38	42	5
**65**	40	47	46	44	4
**66**	47	49	45	47	2
**67**	41	40	48	44	4
**68**	49	46	41	45	4
**70**	41	43	49	44	4
**80**	46	41	40	43	3
**90**	39	40	44	41	3
**100**	40	39	43	41	2
**110**	40	43	40	41	2
**Total Average**				**41**	**4**
**Mn**					
**58**	1.83	1.85	1.73	1.80	0.06
**59**	1.76	1.82	1.80	1.79	0.03
**60**	1.74	1.79	1.81	1.78	0.06
**61**	1.68	1.73	1.70	1.70	0.02
**62**	1.75	1.81	1.70	1.75	0.05
**63**	1.81	1.76	1.88	1.82	0.06
**64**	1.75	1.80	1.86	1.80	0.05
**65**	1.85	1.76	1.71	1.77	0.07
**66**	1.85	1.80	1.71	1.79	0.07
**67**	1.82	1.84	1.75	1.80	0.05
**68**	1.77	1.83	1.84	1.81	0.04
**70**	1.84	1.86	1.78	1.83	0.04

**80**	1.77	1.75	1.84	1.79	0.05
**90**	1.87	1.79	1.76	1.81	0.06
**100**	1.73	1.80	1.82	1.78	0.05
**110**	1.86	1.81	1.78	1.82	0.04
**Total Average**				**1.79**	**0.03**
**Fe**					
**58**	1.24	1.28	1.37	1.30	0.07
**59**	1.31	1.38	1.29	1.33	0.05
**60**	1.30	1.36	1.27	1.31	0.07
**61**	1.24	1.31	1.30	1.28	0.04
**62**	1.29	1.34	1.25	1.29	0.04
**63**	1.33	1.25	1.37	1.32	0.06
**64**	1.36	1.30	1.28	1.31	0.04
**65**	1.33	1.29	1.39	1.34	0.05
**66**	1.24	1.30	1.31	1.28	0.04
**67**	1.31	1.34	1.26	1.30	0.04
**68**	1.37	1.31	1.27	1.32	0.05
**70**	1.26	1.22	1.34	1.27	0.06
**80**	1.35	1.29	1.24	1.29	0.05
**90**	1.32	1.25	1.34	1.30	0.05
**100**	1.35	1.26	1.37	1.33	0.06
**110**	1.21	1.28	1.33	1.27	0.06
**Total Average**				**1.31**	**0.03**
**Cu**					
**58**	2.76	2.69	2.58	2.68	0.09
**59**	2.77	2.56	2.69	2.67	0.11
**60**	2.84	2.67	2.64	2.72	0.09
**61**	2.52	2.78	2.66	2.65	0.13
**62**	2.60	2.68	2.84	2.71	0.12
**63**	2.65	2.68	2.88	2.74	0.12
**64**	2.65	2.55	2.82	2.67	0.14
**65**	2.50	2.61	2.79	2.63	0.15
**66**	2.55	2.78	2.66	2.66	0.11
**67**	2.80	2.49	2.66	2.65	0.15
**68**	2.68	2.51	2.77	2.65	0.13
**70**	2.77	2.60	2.83	2.73	0.12
**80**	2.61	2.78	2.55	2.65	0.12
**90**	2.69	2.50	2.81	2.67	0.16
**100**	2.81	2.60	2.52	2.64	0.15
**110**	2.64	2.83	2.57	2.68	0.13
**Total Average**				**2.67**	**0.03**
**Zn**					
**58**	47.5	44.4	53.5	48.5	4.6
**59**	50.5	41.8	57.5	49.9	7.9
**60**	52.8	42.5	57.2	50.8	4.6

**61**	46.6	57.8	42.8	49.1	7.8
**62**	43.6	46.2	52.8	47.5	4.7
**63**	50.8	54.0	46.6	50.5	3.7
**64**	45.3	49.1	51.1	48.5	2.9
**65**	48.9	45.7	51.6	48.7	2.9
**66**	49.6	53.6	46.5	49.9	3.6
**67**	48.0	51.8	53.3	51.0	2.7
**68**	47.5	49.9	53.2	50.2	2.9
**70**	48.2	49.9	43.9	47.3	3.1
**80**	52.1	50.8	47.1	50.0	2.6
**90**	49.3	48.4	45.5	47.7	2.0
**100**	45.4	51.2	52.2	49.6	3.7
**110**	52.6	50.1	46.1	49.6	3.3
**Total Average**				**49.3**	**1.2**

Table S3. The content of Pb (μg/L) in GR and VSR wines after stabilization.

Day	#1	#2	#3	Average	esd
**GR**					
**58th**	6.2	6.1	5.8	6.0	0.2
**64th**	6.0	5.9	5.5	5.8	0.3
**80th**	4.9	5.1	5.5	5.2	0.3
**110th**	5.6	5.3	5.2	5.4	0.2
**Total average**				**5.6±0.4**	
**VSR**					
**58th**	18.0	17.4	12.5	16.0	3.0
**64th**	13.4	15.5	18.4	15.7	2.5
**80th**	15.4	13.2	17.1	15.2	1.9
**110th**	18.1	13.6	15.6	15.8	2.2
**Total average**				**15.7±0.3**	

Table S4a. Overall relative lowering effects (Δ%, with respect to untreated wines) on the content of selected metals (C_{Na}, C_K, C_{Mg}, C_{Ca}, C_{Mn}, C_{Cu}, C_{Zn}), by soaking swelled CMH2 and CMH10 hydrogels, in GR wine (hydrogel /wine 20 mg dry matter /10 mL, 2g/L; not stirred, in the dark, at 20±1°C, for 48 h). Values (mean ± esd) obtained from three samples, and three replicates each sample; full experimental data sets are reported in Table S4b.

Metal	Hydrogel CMH2	Hydrogel CMH10
Na	0	0
K	4±1	4±1
Mg	21±2	25±3
Ca	12±3	11±3
Mn	7±1	4±1
Cu	26±3	28±5
Zn	9±3	12±2

Table S4b. The content of selected metal ions (mg/L) in GR wines after the treatment with CMH2 and CMH10 hydrogels. Data from three replicates. The data were collected after 24, 48 and 72 h after soaking of the hydrogel.

CMH2	#1	#2	#3	Average	Esd
**Mn**					
Before soaking				1.56	0.02
12 h	1.52	1.47	1.44	1.48	0.04
24 h	1.45	1.43	1.49	1.46	0.03
48 h	1.47	1.44	1.44	1.45	0.02
72 h	1.46	1.45	1.43	1.45	0.01
**Cu**					
Before soaking				0.39	0.01
12 h	0.36	0.32	0.31	0.33	0.03
24 h	0.27	0.31	0.33	0.30	0.03
48 h	0.31	0.26	0.29	0.29	0.03
72 h	0.30	0.28	0.29	0.29	0.01
**Zn**					
Before soaking				0.58	0.02
12 h	0.58	0.54	0.53	0.55	0.03
24 h	0.56	0.55	0.52	0.54	0.02
48 h	0.55	0.52	0.53	0.53	0.01
72 h	0.52	0.55	0.52	0.53	0.02
**CMH10**	**#1**	**#2**	**#3**	**Average**	**Esd**
**Mn**					
Before soaking				1.56	0.02
12 h	1.55	1.51	1.52	1.53	0.02
24 h	1.51	1.49	1.53	1.51	0.02
48 h	1.48	1.51	1.52	1.50	0.02
72 h	1.53	1.49	1.48	1.50	0.03
**Cu**					
Before soaking				0.39	0.01
12 h	0.36	0.32	0.30	0.33	0.03
24 h	0.32	0.29	0.27	0.29	0.03
48 h	0.31	0.26	0.28	0.28	0.03
72 h	0.25	0.30	0.28	0.28	0.03
**Zn**					
Before soaking				0.58	0.02
12 h	0.54	0.57	0.50	0.54	0.04
24 h	0.55	0.52	0.50	0.52	0.03
48 h	0.53	0.49	0.51	0.51	0.02
72 h	0.49	0.51	0.52	0.51	0.02

Table S5a. Overall lowering effects (Δ%, with respect to untreated wines) on the content of selected metals (C_{Mn}, C_{Fe}, C_{Cu}, C_{Zn}) by L-207 and L-208 resins in VSR wines (resin beds/wine 300 mg/15 mL, 20g/L; not stirred, in the dark, at 20±1°C, for 48 h). Values (mean ± esd) obtained from three samples, and three replicates each sample; the full experimental data sets are reported in Table S5b.

**Wine/Resin**	**C_{Mn}**	**C_{Fe}**	**C_{Cu}**	**C_{Zn}**
VSR untreated	1.79±0.03	1.31±0.03	2.67±0.03	49±1
VSR/L-207	0.24±0.01	0.25±0.01	0.24±0.01	26±4
Δ%	87±3	81±3	91±3	47±8
VSR/L-208	0.11±0.02	0.28±0.02	0.25±0.05	40±4
Δ%	94±13	79±5	91±17	18±2

Table S5b. Lowering effects (Δ%, relative to the initial values for stabilized untreated products) and C_M data for triplicates of GR and VSR wines treated on beds of L-207 and L208 ionic exchange resins (20.00 g/L, dark, 20±1°C, 48 h).

VSR/L-207	#1	#2	#3	Average	Esd	Δ%
**Mn**						
Before soaking				1.79	0.03	
s1	0.23	0.23	0.24	0.23	0.01	86.9
s2	0.22	0.23	0.23	0.23	0.01	87.3
s3	0.24	0.25	0.25	0.25	0.01	86.3
**Total average**				**0.24±0.01**		**86.8±0.5**
**Cu**						
Before soaking				2.67	0.03	
s1	0.21	0.22	0.23	0.22	0.01	91.7
s2	0.24	0.26	0.25	0.25	0.01	90.6
s3	0.24	0.25	0.25	0.25	0.01	90.7
**Total average**				**0.240±0.02**		**91.0±0.6**
**Fe**						
Before soaking				1.31	0.03	
s1	0.24	0.22	0.25	0.24	0.01	81.8
s2	0.24	0.25	0.25	0.25	0.01	81.0
s3	0.26	0.27	0.28	0.27	0.01	79.4
**Total average**				**0.252±0.02**		**80.7±1.2**
**Zn**						
Before soaking						
s1	22.8	33.2	22.0	26.0	6.2	46.9
s2	22.2	22.7	31.4	25.4	5.2	48.1
s3	27.3	25.6	28.5	27.1	1.5	44.6
**Total average**				**26.2±0.9**		**46.6±1.8**
**VSR/L-208**	**#1**	**#2**	**#3**	**Average**	**Esd**	**Δ%**
**Mn**						
Before soaking				1.79	0.03	
s1	0.10	0.08	0.12	0.10	0.02	94.4
s2	0.12	0.14	0.14	0.13	0.01	92.5
s3	0.11	0.09	0.12	0.11	0.01	94.0
**Total average**				**0.11±0.02**		**93.7±1.0**
**Cu**						
Before soaking				2.67	0.03	
s1	0.19	0.24	0.26	0.23	0.04	91.4
s2	0.26	0.22	0.32	0.27	0.05	90.0
s3	0.19	0.26	0.29	0.25	0.05	90.8
**Total average**				**0.25±0.02**		**90.7±0.7**

**Fe**						
Before soaking				1.31	0.03	
s1	0.24	0.22	0.25	0.24	0.01	81.8
s2	0.33	0.29	0.36	0.33	0.03	75.1
s3	0.26	0.27	0.28	0.27	0.01	79.4
**Total average**				**0.28±0.04**		**78.7±3.4**
**Zn**						
Before soaking						
s1	35.2	43.8	44.0	41.0	5.0	16.3
s2	38.4	36.7	42.0	39.0	2.7	20.3
s3	44.8	41.2	34.6	40.2	5.2	17.9
**Total average**				**40.1±1.0**		**18.2±2.0**
**GR/L-207**	**#1**	**#2**	**#3**	**Average**	**Esd**	**Δ%**
**Mn**						
Before soaking				1.56	0.02	
s1	0.25	0.24	0.30	0.26	0.03	83.1
s2	0.21	0.26	0.29	0.25	0.04	83.8
s3	0.28	0.30	0.31	0.30	0.02	80.9
**Total average**				**0.27±0.02**		**82.6±1.5**
**Cu**						
Before soaking				0.39	0.01	
s1	0.21	0.22	0.27	0.23	0.03	40.2
s2	0.25	0.19	0.26	0.23	0.04	40.3
s3	0.21	0.22	0.25	0.23	0.02	40.8
**Total average**				**0.23±0.01**		**40.5±0.4**
**Zn**						
Before soaking				0.58	0.02	
s1	0.42	0.46	0.49	0.46	0.03	21.4
s2	0.40	0.44	0.51	0.45	0.05	22.1
s3	0.44	0.50	0.43	0.46	0.04	20.7
**Total average**				**0.46±0.01**		**21.4±0.7**
**GR/L-208**	**#1**	**#2**	**#3**	**Average**	**Esd**	**Δ%**
**Mn**						
Before soaking				1.56	0.02	
s1	0.17	0.20	0.22	0.20	0.03	87.2
s2	0.19	0.21	0.23	0.21	0.02	86.6
s3	0.16	0.20	0.25	0.20	0.04	86.9
**Total average**				**0.20±0.01**		**86.9±0.3**
**Cu**						
Before soaking				0.39	0.01	
s1	0.18	0.13	0.14	0.15	0.03	61.5
s2	0.19	0.14	0.13	0.15	0.03	60.7
s3	0.17	0.14	0.13	0.15	0.02	61.8
**Total average**				**0.15±0.01**		**61.3±0.6**

**Zn**						
Before soaking				0.58	0.02	
s1	0.32	0.35	0.36	0.34	0.02	40.8
s2	0.31	0.34	0.36	0.33	0.03	42.6
s3	0.34	0.30	0.36	0.33	0.03	42.7
**Total average**				**0.34±0.01**		**42.0±1.0**

Table S6. The C_{Na}, C_K, C_{Mg}, C_{Ca}, C_{Mn}, C_{Cu}, C_{Zn} (mg/L) as averaged from three samples, and three replicates (#1,#2,#3) each sample in GR wines after treatments with L-207 (20 g/L), as function of soaking time (1, 3, 6, 18, 21, 24, 30, 42, 48 h) at 20±1°C in the dark, under stirring (see text experimental). The values for t = 0 are for untreated samples.

Sample	Time	#1	#2	#3	Average	Total average
**C_{Na}**						
	0					28.3±0.3
s1	1	26.2	28.8	31.2	28.7±2.5	29.2±2.9
s2		27.8	29.8	33.5	30.4±2.9	
s3		25.0	29.2	31.5	28.6±3.3	
s1	3	24.6	29.3	27.3	27.1±2.4	27.2±2.9
s2		27.5	29.8	33.0	30.1±2.8	
s3		20.7	26.1	26.8	24.5±3.3	
s1	6	26.8	28.8	31.4	29.0±2.3	28.8±2.9
s2		25.7	32.1	31.2	29.7±3.5	
s3		24.6	30.2	28.1	27.6±2.8	
s1	18	26.5	31.3	24.1	27.3±3.7	27.5±3.5
s2		31.1	27.4	25.4	28.0±2.9	
s3		23.4	31.2	27.2	27.3±3.9	
s1	21	22.2	28.5	24.3	25.0±3.2	25.8±3.4
s2		28.8	26.2	22.4	25.8±3.2	
s3		26.6	30.4	23.1	26.7±3.6	
s1	24	22.2	28.5	24.3	25.0±3.2	25.8±3.4
s2		28.8	26.2	22.4	25.8±3.2	
s3		26.6	30.4	23.1	26.7±3.6	
s1	30	23.0	25.8	28.4	25.7±2.7	26.0±3.7
s2		26.6	21.8	29.8	26.1±4.0	
s3		21.4	30.2	26.8	26.1±4.4	
s1	42	20.2	25.3	28.4	24.6±4.1	24.5±4.1
s2		19.5	22.9	28.1	23.5±4.3	
s3		22.0	29.5	24.4	25.3±3.8	
s1	48	21.4	25.2	28.5	25.0±3.5	24.9±3.2
s2		27.8	22.0	25.8	25.2±2.9	
s3		21.9	23.8	27.9	24.5±3.1	
**C_K**						
	0					754±17
s1	1	728	752	782	754±27	750±26
s2		748	778	730	752±24	
s3		714	770	745	743±28	
s1	3	688	724	705	706±18	702±18
s2		686	725	700	704±20	
s3		680	695	714	696±17	
s1	6	656	704	670	677±25	672±24
s2		646	665	698	670±26	
s3		668	692	651	670±21	
s1	18	722	708	686	705±18	706±17

s2		690	702	720	704±15	
s3		700	694	728	707±18	
s1	21	722	760	739	740±19	743±20
s2		746	762	725	744±19	
s3		742	721	767	743±23	
s1	24	695	676	655	675±20	678±17
s2		680	697	668	682±15	
s3		696	678	661	678±17	
s1	30	702	680	662	681±20	684±16
s2		686	703	667	685±18	
s3		695	683	674	684±10	
s1	42	712	689	725	709±18	707±15
s2		701	718	698	706±11	
s3		688	710	721	706±17	
s1	48	615	641	665	640±25	643±20
s2		646	664	623	644±20	
s3		644	656	630	643±13	
**C_{Mg}**						
	0					200±6
s1	1	147	126	136	136±10	137±9
s2		151	127	136	138±12	
s3		134	143	136	138±5	
s1	3	128	125	120	124±4	124±3
s2		125	120	127	124±4	
s3		121	123	126	123±2	
s1	6	123	119	117	120±3	120±3
s2		120	116	123	120±3	
s3		118	120	124	121±3	
s1	18	114	117	121	117±3	117±3
s2		117	114	119	117±2	
s3		113	116	119	116±3	
s1	21	114	116	118	116±2	116±1
s2		115	115	117	116±1	
s3		115	116	117	116±1	
s1	24	112	115	116	114±2	115±3
s2		112	115	118	115±3	
s3		111	115	117	114±3	
s1	30	114	111	116	114±2	114±2
s2		112	115	114	114±2	
s3		110	114	116	114±3	
s1	42	112	114	116	114±2	114±2
s2		114	116	113	114±1	
s3		111	114	116	114±3	
s1	48	111	113	115	113±2	113±2
s2		113	115	111	113±2	
s3		111	113	115	113±2	

**C_{Ca}**						
	0					60±3
s1	1	42	44	43	43±1	43±2
s2		40	44	43	42±2	
s3		41	42	43	42±1	
s1	3	38	36	40	38±2	38±3
s2		36	39	41	39±3	
s3		35	38	41	38±3	
s1	6	35	36	39	37±2	37±2
s2		36	34	39	36±2	
s3		36	36	39	37±1	
s1	18	32	34	36	34±2	34±2
s2		36	34	36	35±1	
s3		31	33	36	33±2	
s1	21	37	33	31	34±3	33±3
s2		33	30	36	33±3	
s3		30	33	36	33±3	
s1	24	28	33	37	33±4	32.6±4.2
s2		32	28	37	32±4	
s3		28	36	32	32±4	
s1	30	29	33	35	32±3	33±3
s2		33	35	37	35±2	
s3		27	36	32	32±4	
s1	42	29	32	34	32±3	32±1
s2		32	29	36	32±4	
s3		28	36	32	32±4	
s1	48	31	32	34	33±1	32±1
s2		31	29	33	32±2	
s3		28	36	30	32±4	
**C_{Mn}**						
	0					1.56±0.02
s1	1	0.59	0.61	0.67	0.62±0.04	0.63±0.05
s2		0.63	0.58	0.68	0.63±0.05	
s3		0.58	0.62	0.67	0.62±0.04	
s1	3	0.48	0.45	0.43	0.45±0.02	0.46±0.03
s2		0.42	0.47	0.48	0.46±0.03	
s3		0.49	0.46	0.44	0.46±0.02	
s1	6	0.37	0.41	0.44	0.41±0.03	0.40±0.05
s2		0.45	0.39	0.36	0.40±0.05	
s3		0.47	0.38	0.36	0.40±0.06	
s1	18	0.34	0.42	0.36	0.37±0.04	0.37±0.04
s2		0.33	0.36	0.41	0.37±0.04	
s3		0.35	0.38	0.42	0.38±0.03	
s1	21	0.42	0.37	0.33	0.37±0.04	0.37±0.04
s2		0.41	0.40	0.33	0.38±0.04	
s3		0.39	0.32	0.39	0.37±0.04	
s1	24	0.38	0.35	0.34	0.37±0.02	0.36±0.02

s2		0.34	0.34	0.38	0.35±0.02	
s3		0.38	0.35	0.34	0.36±0.02	
s1	30	0.37	0.35	0.36	0.36±0.01	0.36±0.02
s2		0.33	0.34	0.38	0.35±0.03	
s3		0.37	0.37	0.34	0.36±0.02	
s1	42	0.36	0.36	0.36	0.36±0.01	0.36±0.02
s2		0.38	0.39	0.33	0.37±0.03	
s3		0.37	0.37	0.34	0.36±0.02	
s1	48	0.37	0.35	0.34	0.35±0.01	0.36±0.03
s2		0.32	0.37	0.38	0.36±0.03	
s3		0.38	0.38	0.31	0.36±0.04	
**C_{Cu}**						
	0					0.39±0.01
s1	1	0.30	0.24	0.26	0.27±0.03	0.27±0.03
s2		0.26	0.25	0.29	0.27±0.02	
s3		0.27	0.30	0.25	0.27±0.02	
s1	3	0.21	0.17	0.20	0.19±0.02	0.20 ±0.02
s2		0.22	0.20	0.19	0.20±0.01	
s3		0.18	0.19	0.21	0.19±0.02	
s1	6	0.12	0.16	0.16	0.15±0.02	0.15±0.01
s2		0.16	0.15	0.15	0.15±0.01	
s3		0.17	0.16	0.16	0.16±0.01	
s1	18	0.13	0.12	0.12	0.12±0.01	0.13±0.01
s2		0.14	0.13	0.14	0.14±0.01	
s3		0.13	0.13	0.15	0.14±0.012	
s1	21	0.13	0.09	0.11	0.11±0.02	0.12±0.02
s2		0.12	0.13	0.13	0.13±0.01	
s3		0.13	0.12	0.09	0.11±0.02	
s1	24	0.12	0.11	0.11	0.11±0.01	0.11±0.01
s2		0.09	0.09	0.11	0.10±0.01	
s3		0.10	0.11	0.11	0.11±0.01	
s1	30	0.11	0.09	0.08	0.10±0.01	0.10±0.02
s2		0.07	0.10	0.12	0.10±0.02	
s3		0.11	0.09	0.11	0.10±0.01	
s1	42	0.10	0.10	0.10	0.10±0.01	0.10±0.01
s2		0.09	0.10	0.12	0.10±0.01	
s3		0.10	0.10	0.12	0.10±0.01	
s1	48	0.09	0.09	0.11	0.10±0.01	0.10±0.01
s2		0.09	0.10	0.11	0.10±0.01	
s3		0.09	0.11	0.09	0.10±0.01	
**C_{Zn}**						
	0					0.58±0.02
s1	1	0.30	0.24	0.37	0.30±0.06	0.31±0.04
s2		0.29	0.32	0.35	0.32±0.03	
s3		0.28	0.31	0.34	0.31±0.03	
s1	3	0.21	0.18	0.23	0.21±0.03	0.20±0.02
s2		0.20	0.19	0.22	0.20±0.01	

s3		0.19	0.18	0.21	0.19±0.01	
s1	6	0.12	0.16	0.13	0.14±0.02	0.12±0.02
s2		0.12	0.13	0.10	0.12±0.02	
s3		0.12	0.10	0.13	0.12±0.02	
s1	18	0.090	0.099	0.093	0.094±0.005	0.093±0.006
s2		0.085	0.094	0.098	0.092±0.007	
s3		0.084	0.100	0.092	0.092±0.008	
s1	21	0.080	0.093	0.085	0.086±0.007	0.086±0.006
s2		0.094	0.082	0.086	0.087±0.006	
s3		0.080	0.091	0.087	0.086±0.006	
s1	24	0.075	0.082	0.088	0.082±0.007	0.081±0.007
s2		0.079	0.074	0.084	0.079±0.005	
s3		0.081	0.089	0.072	0.081±0.009	
s1	30	0.076	0.070	0.061	0.069±0.008	0.069±0.008
s2		0.073	0.060	0.079	0.071±0.010	
s3		0.067	0.075	0.064	0.069±0.006	
s1	42	0.060	0.065	0.056	0.060±0.005	0.058±0.005
s2		0.057	0.052	0.062	0.057±0.005	
s3		0.058	0.051	0.064	0.058±0.007	
s1	48	0.060	0.040	0.053	0.051±0.010	0.047±0.008
s2		0.043	0.045	0.055	0.048±0.006	
s3		0.037	0.042	0.051	0.043±0.007	

Table S7. The C_{Na}, C_K, C_{Mg}, C_{Ca}, C_{Mn}, C_{Fe}, C_{Cu}, C_{Zn} (mg/L) as averaged from three samples, and three replicates (#1,#2,#3) each sample in VSR wines after treatments with L-207 (20 g/L), as function of soaking time (1, 3, 6, 18, 21, 24, 30, 42, 48 h) at 20±1°C in the dark, under stirring (see text experimental). The values for t = 0 are for untreated samples.

Sample	Time	#1	#2	#3	Average	Total average
**C_{Na}**						
	0					20.0±0.5
s1	1	22.2	20.3	18.1	20.2±2.0	20.1±2.2
s2		19.6	23.1	17.8	20.2±2.7	
s3		20.1	18.0	22.0	20.0±2.0	
s1	3	23.1	19.3	21.1	21.2±1.9	21.0±1.8
s2		20.9	22.8	19.5	21.1±1.7	
s3		20.6	19.2	22.7	20.8±1.8	
s1	6	20.0	21.5	18.1	19.9±1.7	19.7±2.3
s2		19.6	17.2	22.1	19.6±2.4	
s3		17.0	19.5	22.3	19.6±2.6	
s1	18	18.0	20.1	23.6	20.6±2.8	20.6±2.9
s2		19.7	18.0	24.1	20.6±3.1	
s3		20.4	18.1	23.8	20.8±2.9	
s1	21	19.7	17.9	24.1	20.6±3.2	20.5±2.8
s2		17.5	20.1	23.0	20.2±2.7	
s3		20.5	18.1	23.2	20.6±2.5	
s1	24	19.0	22.4	18.2	19.9±2.2	20.1±2.3
s2		20.1	22.7	17.8	20.2±2.4	
s3		19.8	22.6	18.2	20.2±2.2	
s1	30	20.8	23.8	17.6	20.7±3.1	20.6±2.7
s2		19.5	18.6	23.1	20.4±2.4	
s3		20.1	18.2	23.4	20.6±2.6	
s1	42	19.9	22.4	18.3	20.2±2.1	20.3±2.4
s2		19.6	17.8	22.4	19.9±2.3	
s3		23.8	19.7	18.4	20.6±2.8	
s1	48	19.0	16.8	22.1	19.3±2.7	19.4±3.0
s2		16.5	18.9	22.8	19.4±3.2	
s3		23.0	16.8	19.1	19.6±3.1	
**C_K**						
	0					685±24
s1	1	650	631	668	650±18	648±20
s2		645	628	670	648±21	
s3		624	649	666	646±21	
s1	3	671	688	710	690±20	690±18
s2		675	708	685	689±17	
s3		705	692	673	690±16	
s1	6	678	701	663	681±19	682±18
s2		699	681	661	680±19	

s3		680	702	669	684±17	
s1	18	652	696	672	673±22	674±19
s2		696	655	679	677±21	
s3		669	688	657	671±16	
s1	21	663	680	648	664±16	666±17
s2		659	682	657	666±14	
s3		661	692	648	667±23	
s1	24	674	628	648	650±23	649±22
s2		622	649	672	648±25	
s3		644	635	670	650±18	
s1	30	703	684	665	684±19	686±20
s2		663	688	705	685±21	
s3		692	707	665	688±21	
s1	42	683	666	697	682±15	681±19
s2		661	681	705	682±22	
s3		702	675	662	680±20	
s1	48	625	644	680	650±289	655±26
s2		652	681	632	655±25	
s3		683	667	633	661±25	
**C_{Mg}**						
	0					174±5
s1	1	135	140	138	138±2	138±3
s2		140	134	137	137±3	
s3		1380	142	135	139±4	
s1	3	127	128	127	128±1	128±2
s2		128	130	125	127±3	
s3		129	128	126	128±2	
s1	6	123	124	121	123±1	123±1
s2		122	123	124	123±1	
s3		123	121	122	122±1	
s1	18	130	125	127	127±2	128±4
s2		128	131	126	128±3	
s3		132	131	120	128±7	
s1	21	125	121	129	125±4	125±2
s2		127	125	123	125±2	
s3		125	126	124	125±1	
s1	24	119	116	123	119±3	119±3
s2		115	116	122	118±4	
s3		121	123	117	120±3	
s1	30	122	118	124	121±3	122±3
s2		123	124	119	122±3	
s3		124	124	120	123±2	
s1	42	116	118	113	116±2	116±2
s2		117	115	115	116±1	
s3		113	115	118	115±2	

s1	48	109	108	106	108±1	107±2
s2		109	107	105	107±2	
s3		107	103	108	106±3	
**C_{Ca}**						
	0					41.1±3.2
s1	1	20.7	22.4	24.2	22.4±1.7	22.5±1.7
s2		22.1	20.5	23.8	22.1±1.6	
s3		21.0	22.8	24.6	22.8±1.8	
s1	3	17.1	18.2	19.1	18.1±1.0	18.1±1.4
s2		17.9	17.2	19.6	18.2±1.2	
s3		15.8	18.9	19.4	18.0±1.9	
s1	6	17.4	15.1	19.3	17.3±2.1	17.0±2.2
s2		14.8	17.6	19.7	17.4±2.5	
s3		18.2	16.4	14.2	16.3±2.0	
s1	18	17.0	14.8	18.5	16.8±1.9	17.1±1.8
s2		17.6	15.1	19.3	17.3±2.1	
s3		17.2	15.8	18.6	17.2±1.4	
s1	21	18.0	16.5	19.3	17.9±1.4	17.7±1.3
s2		17.4	16.1	18.6	17.4±1.2	
s3		16.4	17.6	19.0	17.8±1.3	
s1	24	18.3	19.9	16.0	18.1±2.0	17.9±2.0
s2		20.1	15.7	17.7	17.8±2.2	
s3		17.9	15.9	19.7	17.8±1.9	
s1	30	16.7	15.7	18.6	17.0±1.5	17.3±1.7
s2		15.5	17.4	19.1	17.3±1.8	
s3		15.9	19.6	17.6	17.7±1.8	
s1	42	18.7	17.1	19.3	18.4±1.1	18.2±1.2
s2		18.3	19.5	16.2	18.0±1.7	
s3		19.2	18.1	17.8	18.4±0.7	
s1	48	17.6	16.5	19.3	17.8±1.4	17.8±1.4
s2		16.8	18.2	19.9	18.3±1.5	
s3		17.4	15.9	18.5	17.3±1.3	
**C_{Mn}**						
	0					1.79±0.03
s1	1	0.69	0.78	0.57	0.68±0.10	0.68±0.10
s2		0.80	0.59	0.67	0.69±0.11	
s3		0.65	0.77	0.57	0.66±0.10	
s1	3	0.50	0.54	0.56	0.53±0.03	0.53±0.04
s2		0.54	0.56	0.46	0.52±0.05	
s3		0.55	0.53	0.49	0.52±0.03	
s1	6	0.49	0.51	0.42	0.47±0.05	0.47±0.04
s2		0.53	0.47	0.45	0.48±0.04	
s3		0.45	0.44	0.51	0.47±0.04	
s1	18	0.43	0.46	0.50	0.46±0.03	0.47±0.03
s2		0.47	0.51	0.45	0.48±0.03	

s3		0.48	0.49	0.43	0.47±0.03	
s1	21	0.44	0.45	0.48	0.46±0.02	0.47±0.03
s2		0.46	0.46	0.49	0.47±0.02	
s3		0.46	0.43	0.52	0.47±0.05	
s1	24	0.43	0.48	0.42	0.44±0.03	0.45±0.02
s2		0.45	0.42	0.46	0.44±0.02	
s3		0.47	0.45	0.43	0.45±0.02	
s1	30	0.39	0.47	0.44	0.43±0.04	0.44±0.03
s2		0.41	0.46	0.46	0.44±0.03	
s3		0.45	0.46	0.42	0.44±0.02	
s1	42	0.52	0.48	0.44	0.48±0.04	0.47±0.03
s2		0.48	0.45	0.46	0.46±0.01	
s3		0.44	0.45	0.49	0.46±0.03	
s1	48	0.43	0.42	0.49	0.45±0.04	0.45±0.04
s2		0.40	0.44	0.51	0.45±0.06	
s3		0.45	0.44	0.48	0.46±0.02	
**C_{Fe}**						
	0					1.31±0.03
s1	1	0.44	0.57	0.48	0.50±0.07	0.50±0.07
s2		0.56	0.47	0.46	0.50±0.05	
s3		0.51	0.58	0.43	0.51±0.07	
s1	3	0.33	0.46	0.41	0.40±0.07	0.40±0.05
s2		0.45	0.42	0.36	0.41±0.05	
s3		0.43	0.41	0.35	0.40±0.04	
s1	6	0.37	0.33	0.31	0.34±0.03	0.34±0.03
s2		0.35	0.38	0.31	0.35±0.03	
s3		0.37	0.34	0.31	0.34±0.03	
s1	18	0.26	0.27	0.29	0.27±0.01	0.28±0.02
s2		0.27	0.27	0.30	0.28±0.02	
s3		0.25	0.27	0.30	0.27±0.02	
s1	21	0.23	0.25	0.28	0.25±0.02	0.26±0.02
s2		0.24	0.25	0.28	0.26±0.02	
s3		0.25	0.27	0.26	0.26±0.01	
s1	24	0.20	0.21	0.27	0.23±0.04	0.23±0.03
s2		0.26	0.24	0.22	0.24±0.02	
s3		0.25	0.24	0.21	0.23±0.02	
s1	30	0.21	0.19	0.24	0.21±0.03	0.21±0.03
s2		0.22	0.23	0.19	0.21±0.02	
s3		0.20	0.18	0.25	0.21±0.046	
s1	42	0.17	0.24	0.20	0.20±0.03	0.20±0.03
s2		0.16	0.20	0.26	0.22±0.05	
s3		0.19	0.20	0.21	0.20±0.01	
s1	48	0.21	0.18	0.22	0.20±0.02	0.20±0.02
s2		0.19	0.18	0.20	0.19±0.01	
s3		0.22	0.20	0.19	0.20±0.01	

**C_{Cu}**						
	0					2.68±0.03
s1	1	1.22	1.12	0.97	1.10±0.13	1.10±0.11
s2		1.02	1.11	1.18	1.10±0.08	
s3		0.99	1.06	1.25	1.10±0.13	
s1	3	1.06	0.85	0.71	0.87±0.18	0.89±0.14
s2		0.75	0.89	1.04	0.89±0.14	
s3		0.78	0.92	0.99	0.90±0.11	
s1	6	0.70	0.76	0.53	0.66±0.12	0.67±0.12
s2		0.78	0.69	0.56	0.68±0.11	
s3		0.68	0.79	0.58	0.68±0.10	
s1	18	0.41	0.45	0.37	0.41±0.04	0.41±0.04
s2		0.46	0.42	0.38	0.42±0.04	
s3		0.43	0.39	0.37	0.40±0.03	
s1	21	0.32	0.34	0.39	0.35±0.04	0.35±0.03
s2		0.32	0.37	0.34	0.34±0.02	
s3		0.33	0.35	0.36	0.35±0.01	
s1	24	0.33	0.34	0.37	0.35±0.02	0.35±0.02
s2		0.35	0.36	0.31	0.34±0.03	
s3		0.34	0.36	0.36	0.35±0.01	
s1	30	0.32	0.29	0.33	0.31±0.02	0.31±0.02
s2		0.34	0.31	0.30	0.32±0.02	
s3		0.30	0.31	0.30	0.30±0.01	
s1	42	0.24	0.23	0.28	0.25±0.03	0.25±0.02
s2		0.23	0.25	0.27	0.25±0.02	
s3		0.23	0.26	0.28	0.26±0.02	
s1	48	0.24	0.25	0.24	0.24±0.01	0.25±0.02
s2		0.24	0.23	0.27	0.25±0.02	
s3		0.25	0.23	0.28	0.25±0.02	
**C_{Zn}**						
	0					49.3±1.2
s1	1	5.60	6.20	3.40	5.07±1.47	5.24±1.42
s2		7.00	5.10	4.10	5.40±1.47	
s3		5.00	6.70	4.10	5.27±1.32	
s1	3	1.90	2.06	2.40	2.12±0.25	2.07±0.16
s2		2.03	1.91	2.20	2.05±0.14	
s3		2.03	1.94	2.13	2.03±0.09	
s1	6	1.26	1.32	1.22	1.27±0.05	1.26±0.06
s2		1.19	1.24	1.29	1.24±0.05	
s3		1.25	1.21	1.35	1.27±0.07	
s1	18	1.27	1.21	1.17	1.22±0.05	1.21±0.04
s2		1.19	1.25	1.22	1.22±0.03	
s3		1.17	1.20	1.24	1.20±0.03	
s1	21	1.04	1.11	0.98	1.04±0.06	1.06±0.05
s2		1.09	1.00	1.06	1.05±0.04	

s3		1.02	1.07	1.13	1.07±0.05	
s1	24	1.32	1.39	1.27	1.33±0.06	1.34±0.06
s2		1.36	1.26	1.40	1.34±0.07	
s3		1.28	1.35	1.39	1.34±0.05	
s1	30	1.05	0.96	0.98	1.00±0.05	1.00±0.06
s2		0.96	1.01	1.03	1.00±0.04	
s3		0.93	1.03	1.10	1.02±0.08	
s1	42	0.91	0.91	0.96	0.93±0.03	0.93±0.006
s2		0.99	0.92	0.87	0.93±0.06	
s3		0.96	1.06	0.87	0.96±0.09	
s1	48	1.01	1.13	0.97	1.04±0.08	1.06±0.08
s2		1.14	1.04	0.99	1.06±0.08	
s3		1.01	1.07	1.15	1.08±0.07	

Table S8. Trend for pH values as averaged from three samples, and three replicates (#1,#2,#3) each sample in GR and VSR wines after treatments with L-207 (20 g/L), as function of soaking time (1, 3, 6, 18, 21, 24, 30, 42, 48 h) at 20±1°C in the dark, under stirring (see text experimental). The values for t = 0 are for untreated samples.

Sample	Time	#1	#2	#3	Average	Total average
**GR**						
s1	0	3.51	3.46	3.44	3.47±0.04	3.47±0.03
s2		3.47	3.44	3.48	3.46±0.02	
s3		3.52	3.48	3.46	3.49±0.03	
s1	6	3.34	3.32	3.29	3.32±0.02	3.32±0.02
s2		3.31	3.31	3.34	3.32±0.02	
s3		3.30	3.32	3.32	3.31±0.01	
s1	18	3.33	3.36	3.29	3.33±0.03	3.33±0.02
s2		3.35	3.34	3.32	3.34±0.01	
s3		3.31	3.32	3.32	3.32±0.01	
s1	24	3.29	3.30	3.34	3.31±0.03	3.32±0.03
s2		3.33	3.30	3.35	3.33±0.02	
s3		3.28	3.33	3.33	3.31±0.03	
s1	36	3.30	3.31	3.34	3.32±0.02	3.31±0.02
s2		3.29	3.30	3.33	3.31±0.02	
s3		3.35	3.31	3.30	3.32±0.03	
s1	48	3.32	3.32	3.33	3.32±0.01	3.32±0.01
s2		3.29	3.30	3.34	3.31±0.03	
s3		3.33	3.32	3.31	3.32±0.01	
**VSR**						
s1	0	3.43	3.46	3.47	3.45±0.02	3.45±0.02
s2		3.48	3.42	3.45	3.45±0.03	
s3		3.41	3.44	3.46	3.44±0.02	
s1	6	3.2	3.23	3.26	3.23±0.03	3.24±0.03
s2		3.22	3.25	3.26	3.24±0.02	
s3		3.21	3.24	3.27	3.24±0.03	
s1	18	3.24	3.23	3.24	3.24±0.01	3.24±0.02
s2		3.21	3.22	3.26	3.23±0.03	
s3		3.25	3.25	3.22	3.24±0.02	
s1	24	3.22	3.23	3.26	3.24±0.02	3.23±0.02
s2		3.21	3.24	3.25	3.23±0.02	
s3		3.2	3.25	3.24	3.23±0.03	
s1	36	3.18	3.2	3.23	3.20±0.02	3.21±0.02
s2		3.22	3.19	3.23	3.21±0.02	
s3		3.2	3.21	3.22	3.21±0.01	
s1	48	3.22	3.21	3.25	3.23±0.02	3.22±0.02
s2		3.19	3.21	3.24	3.21±0.02	
s3		3.2	3.21	3.23	3.21±0.01	

Table S9. Trend for color index (CI, Abs, λ 420 nm) values as averaged from three samples, and three replicates (#1,#2,#3) each sample in GR and VSR wines after treatments with regenerated L-207 (20 g/L), as function of soaking time (1, 3, 6, 18, 21, 24, 30, 42, 48 h) at 20±1°C in the dark, under stirring (see text experimental). The values for t = 0 are for untreated samples.

Sample	Time	#1	#2	#3	Average	Total average
**GR**						
s1	0	0.431	0.438	0.444	0.438±0.007	0.437±0.007
s2		0.428	0.436	0.445	0.436±0.009	
s3		0.442	0.430	0.440	0.436±0.006	
s1	6	0.380	0.369	0.355	0.368±0.013	0.366±0.012
s2		0.372	0.377	0.353	0.367±0.013	
s3		0.371	0.351	0.369	0.364±0.011	
s1	18	0.370	0.381	0.358	0.370±0.012	0.372±0.012
s2		0.376	0.363	0.384	0.374±0.011	
s3		0.372	0.361	0.390	0.373±0.013	
s1	24	0.367	0.360	0.375	0.367±0.008	0.367±0.007
s2		0.358	0.366	0.377	0.367±0.010	
s3		0.369	0.361	0.371	0.367±0.005	
s1	36	0.365	0.360	0.370	0.365±0.005	0.364±0.006
s2		0.367	0.361	0.359	0.362±0.004	
s3		0.363	0.356	0.372	0.364±0.008	
s1	48	0.368	0.359	0.372	0.366±0.007	0.366±0.008
s2		0.355	0.366	0.375	0.365±0.010	
s3		0.361	0.369	0.373	0.368±0.006	
**VSR**						
s1	0	1.040	1.055	1.067	1.054±0.014	1.056±0.014
s2		1.042	1.060	1.068	1.057±0.014	
s3		1.043	1.074	1.06	1.059±0.016	
s1	6	0.902	0.910	0.919	0.910±0.009	0.912±0.009
s2		0.914	0.921	0.904	0.913±0.009	
s3		0.902	0.910	0.922	0.911±0.010	9
s1	18	0.905	0.897	0.911	0.904±0.007	0.903±0.00
s2		0.892	0.901	0.912	0.902±0.010	
s3		0.890	0.906	0.910	0.902±0.011	
s1	24	0.907	0.899	0.913	0.906±0.007	0.906±0.008
s2		0.899	0.912	0.905	0.905±0.007	
s3		0.914	0.905	0.896	0.905±0.009	
s1	36	0.900	0.909	0.893	0.901±0.008	0.901±0.007
s2		0.911	0.899	0.895	0.902±0.008	
s3		0.908	0.901	0.896	0.902±0.006	
s1	48	0.903	0.906	0.899	0.903±0.003	0.902±0.006
s2		0.909	0.901	0.894	0.901±0.008	
s3		0.907	0.902	0.896	0.902±0.006	

Table S10. Selected instrumental parameters and calibration data for analyzed metal ions.

**Element**	**Lamp wavelength**	**Calibration range**	**Equation // R^2**	**LOQ// LOD**
Na	589.0 nm	0.10–2.50 mg/L	y=0.2315x // 0.9999	0.100 // 0.030 mg/L
K	766.5 nm	0.20–5.00 mg/L	y=0.1361x // 0.9998	0.200 // 0.070 mg/L
Mg	285.2 nm	0.05–1.00 mg/L	y=0.5644x // 0.9960	0.050 // 0.020 mg/L
Ca	422.7 nm	0.50–6.00 mg/L	y=0.0439x // 0.9996	0.300 // 0.100 mg/L
Cu	324.7 nm	0.10–1.00 mg/L	y=0.0555x // 0.9999	0.100 // 0.030 mg/L
Mn	279.5 nm	0.30–4.00 mg/L	y=0.0728x // 0.9997	0.100 // 0.030 mg/L
Zn	213.9 nm	0.20–2.50 mg/L	y=0.2679x // 0.9990	0.050 // 0.020 mg/L
Fe	248.3 nm	0.50–8.00 mg/L	y=0.0283x // 0.9999	0.200 // 0.070 mg/L
Pb	217.0 nm	1.00–10.00 µg/L	y=0.2587x // 0.9890	1.00 // 0.30 µg/L
